# Obesity, psychological well-being related measures, and risk of seven non-communicable diseases: evidence from longitudinal studies of UK and US older adults

**DOI:** 10.1038/s41366-024-01551-1

**Published:** 2024-06-01

**Authors:** I Gusti Ngurah Edi Putra, Michael Daly, Angelina Sutin, Andrew Steptoe, Shaun Scholes, Eric Robinson

**Affiliations:** 1 Department of Psychology, Institute of Population Health, University of Liverpool, Liverpool, UK; 2 Department of Psychology, Maynooth University, Maynooth, Ireland; 3 Department of Behavioral Sciences and Social Medicine, Florida State University College of Medicine, Tallahassee, FL USA; 4 Department of Epidemiology and Health, Institute of Epidemiology and Health Care, Faculty of Population Health Sciences, University College London, London, UK

**Keywords:** Risk factors, Epidemiology, Cardiovascular diseases

## Abstract

**Background:**

We examined the role of psychological well-being related measures in explaining the associations between obesity and increased risk of non-communicable diseases (NCDs: hypertension, heart disease, stroke, diabetes, arthritis, cancer, and memory-related disease) in older adults.

**Methods:**

Data were from the English Longitudinal Study of Ageing (ELSA), UK (baseline: Wave 4—2008/2009; *n* = 8127) and the Health and Retirement Study (HRS), US (baseline: Waves 9 and 10—2008/2010; *n* = 12,477). Objective body mass index was used to define obesity. A range of psychological well-being related measures (e.g., depressive symptoms, life satisfaction) was available in ELSA (*n* = 7) and HRS (*n* = 15), and an index of overall psychological well-being was developed separately in each study. NCDs were from a self-reported doctor diagnosis and/or other assessments (e.g., biomarker data) in both studies; and in ELSA, NCDs from linked hospital admissions data were examined. Longitudinal associations between obesity status, psychological well-being measures, and NCDs were examined using Cox proportional hazard models (individual NCDs) and Poisson regression (a cumulative number of NCDs). Mediation by psychological well-being related measures was assessed using causal mediation analysis.

**Results:**

Obesity was consistently associated with an increased prospective risk of hypertension, heart disease, diabetes, arthritis, and a cumulative number of NCDs in both ELSA and HRS. Worse overall psychological well-being (index measure) and some individual psychological well-being related measures were associated with an increased prospective risk of heart disease, stroke, arthritis, memory-related disease, and a cumulative number of NCDs across studies. Findings from mediation analyses showed that neither the index of overall psychological well-being nor any individual psychological well-being related measures explained (mediated) why obesity increased the risk of developing NCDs in both studies.

**Conclusion:**

Obesity and psychological well-being may independently and additively increase the risk of developing NCDs.

## Introduction

Obesity is now a major health problem in many countries. In England, the prevalence of obesity almost doubled from 15 to 28% between 1993–2019 [1]. The prevalence of obesity in the US is higher at 42% in 2017–2020 which increased from 31% in 1999–2000 [2]. Obesity is associated with an increased risk of numerous non-communicable diseases (NCDs), including cardiovascular diseases, diabetes mellitus, musculoskeletal disorders, and cancer [3], as well as shortened life expectancy by up to 14 years [4]. Due to the reported negative impacts of obesity, understanding the mechanisms which explain why obesity increases risks of NCDs will be key to prevention efforts and reducing the obesity-related NCD burden.

Adverse physical health outcomes of obesity have been traditionally explained by the objective effect of adiposity (i.e., excess fat mass) on a range of physiological processes in the body. Accumulation of visceral adipose tissue (i.e., adipose tissue localised intra-abdominally in visceral compartments) is thought to be responsible for metabolic dysregulations, including inflammation, dyslipidaemia, and insulin resistance [5, 6]. In line with this hypothesis, empirical studies found that obesity is associated with a range of clinical indicators of metabolic (e.g., dyslipidaemia) [7], cardiovascular (e.g., heart rates) [8], and immune system functioning [9], including when these indicators were combined into a ‘physiological dysregulation’ index [10, 11]. Physiological effects of obesity have been shown to increase the risk of developing disease. For example, dyslipidaemia is associated with an increased risk of heart disease [12], cancer [13], and diabetes [14]. In addition, obesity is a major factor associated with C-reactive protein (CRP) concentration, a marker of systemic inflammation in rheumatoid arthritis [15]. Elevated CRP levels may also increase the risk of dementia [16].

Recent psychosocial models of obesity suggest that impaired psychological well-being and negative feelings associated with heavier body weight may explain some of the health consequences of living with obesity [17, 18]. Many people living with obesity experience weight stigma [19] that can negatively contribute to psychological well-being related outcomes, such as depressive symptoms [20]. Other adverse psychological well-being outcomes are reported more commonly in people living with obesity (vs. without), including anxiety [21], loneliness [22], and low life satisfaction [23]. There is also some evidence that compromised psychological well-being (e.g., depressive symptoms, anxiety) is associated with future NCD risk, including hypertension [24], heart disease [25], stroke [26], diabetes [27], arthritis [28], cancer [29], and dementia [30]. This may occur through physiological and/or behavioural mechanisms. For instance, depressive symptoms are associated with elevated CRP levels, metabolic syndrome [31, 32], and health risk behaviours (e.g., unhealthy eating, physical inactivity) [33, 34] that are known risk factors for NCDs.

To date, whether the NCD risk obesity causes is explained (mediated) by the psychological burden of obesity and/or whether obesity and psychological well-being related measures have independent and additive contributions to risk of developing NCDs has yet to be tested formally. Our previous study assessed the role of psychological well-being related measures in explaining the prospective association between obesity and physiological health, which provided some evidence that obesity-related declines in health biomarkers were explained partly by the psychological burden of being discriminated against due to body weight in UK and US older participants [10]. However, the extent to which psychological well-being related measures explain obesity-related NCD risk has not been investigated.

In this present study, we examined the role of a broad range of psychological well-being related measures in explaining the prospective association between obesity and seven NCDs (hypertension, heart disease, stroke, diabetes, arthritis, cancer, and memory-related disease). Consistent with theoretical models which suggest that the psychosocial burden of obesity may contribute to obesity-related disease outcomes, we hypothesised that psychological well-being related measures may explain why obesity is associated with increased NCD risk. We used two large comparable representative longitudinal studies from the UK and the US. We first tested our hypotheses using data from the English Longitudinal Study of Ageing (ELSA), UK and then examined the generalisability of the findings to the US; Health and Retirement Study (HRS).

## Methods

The analysis approach for this study was pre-registered at 10.17605/OSF.IO/JRQAP.

### Study design and data

#### English Longitudinal Study of Ageing (ELSA)

ELSA is a representative longitudinal study of English non-institutional older adults (≥50 years). ELSA commenced in 2002 (Wave 1) and follow-up interviews have been conducted every 2 years from 2004–2005 (Wave 2) to 2021–2023 (Wave 10). Physical and biomarker assessments were carried out for all participants every 4 years (Wave 2, 4, 6, 8/9) [35]. For this study, we selected Wave 4 (2008–2009) as a baseline because a large range of psychological well-being related measures was collected in this wave. Follow-up assessments on NCDs ranged from Wave 5 up to the most recent available data (Wave 9) with a maximum follow-up period of 10 years. The maximum analytical sample size for the analysis using ELSA was *n* = 8127.

#### Health and Retirement Study (HRS)

We replicated the analyses of ELSA using HRS, a comparable representative longitudinal study of older adults in the US, that collected a wider range of psychological well-being related measures. HRS recruited participants in 1992 (Wave 1) with biennial follow-ups conducted from 1994 (Wave 2) to 2020 (Wave 15). From 2006 (Wave 8), HRS expanded its content by including a set of biomarker, physical, and psychosocial assessments through a mixed-mode design for follow-up. Half of the participants were randomly selected for the first enhanced face-to-face interview (EFTF) at Wave 8 for the expanded content and the other half only participated in the core interview. From Wave 9, the data collection method was swapped between the two half-sample groups [36]. For our study, we combined the two half EFTF samples from Wave 9 (2008) and Wave 10 (2010) as a baseline because psychological well-being related measures were collected using the same tools. Follow-up assessments of NCDs started from Waves 10 and 11 for participants with baseline measures at Waves 9 and 10, respectively, and up to Wave 15 with a maximum follow-up period of 10 years. The maximum analytical sample size was *n* = 12,477.

### Variables

#### Exposure: obesity

An objective measure of body mass index (BMI) in kg/m^2^ was used to define obesity at baseline in ELSA and HRS (see Supplementary Materials for more information). Following previous studies [37, 38], our analysis mainly compared the risk of developing an NCD between participants with obesity (BMI ≥ 30) and normal weight (BMI 18.5–24.9). If we found no evidence of relative risk based on this comparison, we compared Class II & III obesity (BMI ≥ 35) with normal weight. We excluded participants with underweight (BMI < 18.5) as some diseases can cause weight loss and then may increase the risk of ill health in participants with underweight [39].

#### Outcomes: non-communicable diseases (NCDs)

Following a recently published study that examined multimorbidity based on 14 health outcomes available in ELSA [40], our study selected seven health outcomes or NCDs that have been found to be associated with both obesity and psychological well-being related measures: hypertension, heart disease, stroke, diabetes mellitus, arthritis, cancer, and dementia or memory-related disease (see Table A1 in Supplementary Materials). For mediation analyses, we examined NCDs that were predicted by obesity at baseline. Most NCDs were defined based on self-reported doctor diagnosis only, whilst some combined a self-reported doctor-diagnosis measure with other assessments (e.g., blood pressure for hypertension and fasting plasma glucose or HbA1c for diabetes). Table A2 presents how each NCD was determined in ELSA and HRS.

#### Candidate mediators: psychological well-being related measures

Table A3 presents a list of psychological well-being related measures available at baseline in ELSA (*n* = 7) and HRS (*n* = 15). Five psychological well-being related measures in ELSA were also available for analysis in HRS (see Supplementary Material for full information). All the psychological well-being related measures were standardised using *z*-scores to allow comparison across different metrics.

For the main analysis, we developed a composite index of overall psychological well-being measures separately in ELSA and HRS. Following our previous approach [41], we used exploratory factor analysis (EFA) to retain psychological well-being related measures that loaded onto the same underlying single factor, defined as having an item loading ≥0.55 [42]. In ELSA, five psychological well-being related measures were combined into an index (depressive symptoms, enjoyment of life, eudemonic well-being, life satisfaction, and loneliness). In HRS, ten psychological well-being related measures were combined into an index (depressive symptoms, life satisfaction, loneliness, positive affect, negative affect, purpose in life, anxiety, hopelessness, pessimism, and personal constraint, otherwise known as lack of self-control). Some psychological well-being related measures (e.g., enjoyment of life, eudemonic well-being, life satisfaction) were reverse scored to be consistent with other measures that were in the direction of lower psychological well-being (e.g., depressive symptoms) and then *z*-score transformed. This index of overall psychological well-being was calculated separately in each study by re-standardising average standardised scores of the retained psychological well-being related measures. A higher index indicated lower overall psychological well-being.

Following evidence of a statistically significant association between obesity and an NCD, mediation by the index of overall psychological well-being measures was tested in the main analysis. The benefit of this index approach is that it provides a more complete picture of overall psychological well-being in individual participants and addresses problems associated with examining a large number of measures in concert (e.g., multi-collinearity, model overfitting). However, additional mediation analyses for individual psychological well-being related measures that met preconditions for successful mediation were also conducted. We included an individual psychological well-being related measure if that measure was associated with both obesity and an NCD.

#### Covariates

Sociodemographic covariates that were controlled in the analysis included age (in years), sex (male, female), paid employment status (yes, no), ethnicity (White, non-White), marital status (single/never married, married/cohabiting, and separated/divorced/widowed), and educational level: the completion of degree-level qualification (yes; no) in ELSA (e.g., as in [11]) and the total number of years in education in HRS (e.g., as in [10]). We also controlled total household wealth (net non-pension wealth) in both studies that was transformed into quintiles (e.g., as in [10]).

### Data analysis

#### Main analyses

We used Cox proportional hazard regression models to examine the associations between obesity at baseline and the incidence of each NCD, controlling for sociodemographic covariates. Survival time was calculated from the baseline interview date to the date of diagnosis or follow-up interview when an NCD was reported for the first time after the baseline (e.g., as in [43]). For each regression model predicting an NCD, participants who had that condition before or at baseline were excluded. We also used Cox models to evaluate the associations between the index of overall psychological well-being, individual psychological well-being related measures at baseline, and the incidence of NCDs. Findings were presented as hazard ratios (HR) along with 95% confidence intervals (CI) and *p* values. In addition, linear regression models were used to examine the cross-sectional association between obesity and psychological well-being related measures as part of pre-mediation condition analyses. Regression coefficient (*β*), 95% CI, and *p* values were presented as the results.

We assessed mediation by an index of overall psychological well-being if we found any evidence of the association between obesity and an NCD. We used causal mediation analysis to conduct mediation analysis (see Supplementary Materials for full information). Findings were presented as the overall proportion of the association due to mediation along with 95% CI and *p* values. Supplementary mediation analyses by each individual psychological well-being related measure were conducted if preconditions for successful mediation were satisfied. To address potential selection bias due to missing observations, we used the inverse probability weighting approach [44, 45] (see Supplementary Materials for full information).

We set *p* < 0.05 for the main regression analysis of the associations between obesity, index of overall psychological well-being, and NCDs, and mediation analysis by the index. To correct for multiple comparisons, *p* < 0.01 was set for supplementary analyses for individual psychological well-being related measures, including regression analyses for examining preconditions for successful mediation (associations between individual psychological well-being related measures and (1) obesity and (2) NCDs) and single mediation analyses by each psychological well-being related measure. The same significance level (*p* < 0.01) was also applied for the following additional analyses.

#### Additional analyses

We conducted additional analyses, including the outcome assessed as the total number of NCDs identified during follow-up in both studies and comparative analysis for ELSA by linking ELSA with hospital admissions data (Hospital Episode Statistics). To better understand potential independent vs. related effects of psychological well-being related measures and body weight on NCDs, we also explored if weight change (measured as change in BMI) between waves in part explained any prospective associations between individual psychological well-being related measures at baseline and NCDs (see Supplementary Materials for full information). We also conducted (non-preregistered) interaction analyses between obesity status and index of overall psychological well-being related measures in predicting NCDs in both studies.

## Results

### Characteristics of participants

Table 1 shows that participants from ELSA and HRS were comparable in terms of age and sex. The proportions of participants who were non-White and had obesity were higher in HRS than in ELSA. HRS participants had higher prevalence of all NCDs at baseline vs. ELSA participants. Likewise, the incidence of NCDs was generally higher in HRS during the follow-up period.

**Table 1:** Characteristics of participants in ELSA and HRS.

Variables	ELSA (*n* = 8127)			HRS (*n* = 12,477)		
	*n*^a^	Mean (SD)^b^	%^b^	*n*^a^	Mean (SD)^b^	%^b^
Baseline characteristics						
Age (years)	8127	65.92 (10.67)		12,477	65.99 (10.15)	
Sex	8127			12,477		
Female			53.45			54.85
Male			46.55			45.15
Ethnicity	8124			12,472		
Non-White			3.76			13.09
White			96.24			86.91
BMI baseline (in kg/m^2^)	7768	28.44 (5.30)		10,906	29.65 (5.98)	
Normal weight			25.97			21.67
Overweight			41.79			36.37
Class I obesity			21.54			25.05
Class II & III obesity			10.70			16.91
The prevalence of NCDs at the baseline^c^						
Hypertension	8126			12,477		
Yes			58.53			69.46
Heart disease	8122			12,477		
Yes			21.57			26.33
Stroke	8122			12,477		
Yes			4.63			7.80
Diabetes mellitus	8126			12,477		
Yes			13.54			24.82
Arthritis	8122			12,476		
Yes			39.00			62.70
Cancer	8122			12,477		
Yes			8.50			15.10
Memory-related disease	8123			12,477		
Yes			1.19			7.28
The incidence of NCDs during the follow-up period^d^						
Hypertension	3097			3113		
Yes			31.19			35.48
Heart disease	5891			8404		
Yes			17.42			18.20
Stroke	7168			10,719		
Yes			4.61			6.91
Diabetes mellitus	6524			8571		
Yes			7.57			13.61
Arthritis	4530			3889		
Yes			21.28			33.16
Cancer	6848			9820		
Yes			9.08			11.99
Memory-related disease	7422			10,773		
Yes			7.69			11.12

^a^Number of participants without missing observations included in the descriptive analysis for each variable (analytical sample size).

^b^Values were weighted using weights from physical assessments (e.g., BMI).

^c^The prevalence was calculated as the number of participants reporting an NCD before and at the baseline (numerator) divided by the number of participants at baseline with no missing information on that NCD (denominator).

^d^The incidence was calculated as the number of participants reporting an NCD during the follow-up period (numerator) divided by the number of participants *without* (1) that condition reported before and at the baseline and (2) missing information on that condition at follow-up (denominator).

### Findings from the English Longitudinal Study of Ageing (ELSA)

Table 2 shows that obesity (vs. normal weight) was associated with increased risk of hypertension (HR = 1.52; 95% CI = 1.25, 1.85), heart disease (HR = 1.45; 95% CI = 1.19, 1.76), diabetes (HR = 4.23; 95% CI = 2.98, 6.01), and arthritis (HR = 1.82; 95% CI = 1.49, 2.23), and these association were also independent of the index of overall psychological well-being. No risk differences in developing stroke, cancer, and memory-related disease were observed between participants with obesity vs. normal weight (Models 1 and 2), or between those with Class II & III obesity vs. normal weight (Table B1).

**Table 2:** Longitudinal associations between obesity (vs. normal weight) and the incidence of NCDs in ELSA.

Outcomes	Obesity vs. normal weight				
	*n*	Model 1		Model 2	
		HR	95% CI	HR	95% CI
Hypertension	2699	1.52	1.25, 1.85***	1.50	1.24, 1.83***
Heart disease	5082	1.45	1.19, 1.76***	1.45	1.19, 1.76***
Stroke	6181	1.19	0.85, 1.65	1.18	0.85, 1.63
Diabetes	5629	4.23	2.98, 6.01***	4.23	2.98, 6.01***
Arthritis	3929	1.82	1.49, 2.23***	1.81	1.48, 2.22***
Cancer	5870	1.21	0.96, 1.53	1.21	0.95, 1.53
Memory-related disease	6376	0.91	0.68, 1.22	0.92	0.69, 1.22

Model 1: the association between obesity and an NCD was adjusted for age, sex, ethnicity, marital status, paid employment status, completion of degree qualification level, and wealth index. Model 2: Model 1 with an additional adjustment for *an index of overall psychological well-being*. Index of overall psychological well-being measures was developed by re-standardising the average standardised scores of five psychological well-being related measures (depressive symptoms, enjoyment of life, eudemonic well-being, life satisfaction, and loneliness). Comparisons between overweight vs. normal weight for each NCD are not presented.

*n* analytical sample size (excluding participants with the outcome before and at baseline and missing observations), *HR* hazard ratio, *CI* confidence interval.

*p < 0.05; **p < 0.01; ***p < 0.001.

Table 3 indicates that index of overall psychological well-being was associated with risk of hypertension, heart disease, stroke, arthritis, and memory-related disease, independently of obesity status. Individual psychological well-being related measures (e.g., depressive symptoms, enjoyment of life) were also independently associated with those NCDs (*p* < 0.01) (Table B2). We found stronger associations between obesity and psychological well-being related measures when Class II & III obesity and normal weight were compared (Table B3), as opposed to when all obesity and normal weight were compared.

**Table 3:** Longitudinal associations between an index of overall psychological well-being and the incidence of NCDs in ELSA.

Outcomes	Index of overall psychological well-being measures				
	*n*	Model 1		Model 2	
		HR	95% CI	HR	95% CI
Hypertension	2699	1.13	1.04, 1.23**	1.12	1.03, 1.22**
Heart disease	5082	1.17	1.09, 1.25***	1.16	1.08, 1.25***
Stroke	6181	1.16	1.03, 1.30*	1.15	1.02, 1.29*
Diabetes	5629	1.06	0.94, 1.19	1.05	0.94, 1.18
Arthritis	3929	1.26	1.17, 1.36***	1.26	1.17, 1.36***
Cancer	5870	1.06	0.95, 1.17	1.05	0.94, 1.16
Memory-related disease	6376	1.43	1.29, 1.58***	1.43	1.29, 1.58***

Model 1: the association between index of overall psychological well-being and an NCD was adjusted for age, sex, ethnicity, marital status, paid employment status, completion of degree qualification level, and wealth index. Model 2: Model 1 with an additional adjustment for *obesity status*. Index of overall psychological well-being measures was developed by re-standardising the average standardised scores of five psychological well-being related measures (depressive symptoms, enjoyment of life, eudemonic well-being, life satisfaction, and loneliness). Comparisons between overweight vs. normal weight for each NCD are not presented.

*n* analytical sample size (excluding participants with the outcome before and at baseline and missing observations), *HR* hazard ratio, *CI* confidence interval.

*p < 0.05; **p < 0.01; ***p < 0.001.

We conducted mediation analyses for NCDs that were associated with obesity. As our findings indicated stronger associations between Class II & III obesity (vs. normal weight) and psychological well-being related measures, mediation analyses were conducted comparing both obesity vs. normal weight, and Class II & III obesity vs. normal weight. However, findings from causal mediation analysis consistently indicated no evidence of mediation by the index of overall psychological well-being (Table 4) or by individual psychological well-being related measures of which preconditions for mediation analysis were satisfied (Table B4).

**Table 4:** Mediation by an index of overall psychological well-being in the longitudinal associations between obesity and the incidence of NCDs in ELSA.

Outcomes	Mediation by an index of overall psychological well-being measures					
	Obesity vs. normal weight			Obesity class II & III vs. normal weight		
	*n*	Estimate	95% CI	*n*	Estimate	95% CI
Hypertension	1532	0.003	−0.018, 0.024	1107	0.024	−0.048, 0.095
Heart disease	2925	0.034	−0.024, 0.092	1885	0.043	−0.008, 0.094
Diabetes	3181	0.001	−0.003, 0.005	2050	−0.002	−0.009, 0.005
Arthritis	2203	0.002	−0.022, 0.027	1452	0.026	−0.027, 0.080

All the mediation analyses were adjusted for age, sex, ethnicity, marital status, paid employment status, completion of degree qualification level, and wealth index. Index of overall psychological well-being was developed by re-standardising the average standardised scores of five psychological well-being related measures (depressive symptoms, enjoyment of life, eudemonic well-being, life satisfaction, and loneliness). As only a binary or continuous independent variable is allowed, causal mediation analysis excluded participants with overweight for the comparison between obesity vs. normal weight, and participants with overweight and Class I obesity for the comparison between Class II & III obesity vs. normal weight, resulting in a smaller analytical sample size compared to previous regression analyses that allowed an ordinal independent variable to be fitted.

*n* analytical sample size (excluding participants with the outcome before and at baseline and missing observations), *Estimate* the overall proportion due to mediation or the proportion mediated, *CI* confidence interval.

*p < 0.05; **p < 0.01; ***p < 0.001

Obesity (vs. normal weight) was also associated with the cumulative number of NCDs across the follow-up period (IRR = 1.35; 95% CI = 1.26, 1.44) (Table B5). For psychological well-being related measures, none were associated with the cumulative number of NCDs at *p* < 0.01 (Table B6). No mediation analysis was conducted for the number of NCDs because not all preconditions for successful mediation were satisfied (see Tables B3, B5 and B6 for more information). Furthermore, we did not find associations between psychological well-being related measures and weight change (Table B7) and weight change were not associated with the incidence of any NCDs (Table B8), and therefore, mediation by weight change was not examined. When the incidence of NCDs in ELSA was classified on the basis of hospital admissions data, results were largely consistent with obesity being a risk factor for NCD incidence but there being no mediation by psychological well-being related measures; obesity (vs. normal weight) was associated with hypertension (HR = 2.23; 95% CI = 1.63, 3.04) and diabetes incidence (HR = 3.80; 95% CI = 2.38, 6.09) with no evidence of mediation (Tables B9–B17). As we found no evidence for mediation, we conducted interaction analyses using survey data and found that the index of overall psychological well-being did not moderate most associations between obesity and NCDs. The exception was memory-related disease (Table B18), whereby the association between obesity status and disease development risk was higher among participants with lower overall psychological well-being (a higher index score).

### Findings from the Health and Retirement Study (HRS)

Similar to ELSA, obesity (vs. normal weight) was associated with increased risk of hypertension (HR = 1.43, 95% CI = 1.18, 1.74), heart disease (HR = 1.27, 95% CI = 1.07, 1.51), diabetes (HR = 2.79, 95% CI = 2.22, 3.51), and arthritis (HR = 1.42, 95% CI = 1.18, 1.70), and these association were independent of the index of overall psychological well-being (Table 5). While there were no risk differences in developing stroke and cancer between participants with obesity vs. normal weight, participants with obesity tended to have lower risk of developing memory-related disease. For most of the NCDs, findings remained when Class II & III obesity and normal weight were compared (Table C1).

**Table 5:** Longitudinal associations between obesity (vs. normal weight) and the incidence of NCDs in HRS.

Outcomes	Obesity vs. normal weight				
	*n*	Model 1		Model 2	
		HR	95% CI	HR	95% CI
Hypertension	2773	1.43	1.18, 1.74***	1.43	1.18, 1.73***
Heart disease	7443	1.27	1.07, 1.51**	1.27	1.07, 1.51**
Stroke	9440	1.17	0.93, 1.49	1.17	0.92, 1.48
Diabetes	7594	2.79	2.22, 3.51***	2.79	2.22, 3.50***
Arthritis	3486	1.42	1.19, 1.70***	1.44	1.20, 1.73***
Cancer	8609	1.21	0.99, 1.48	1.20	0.98, 1.47
Memory-related disease	9462	0.67	0.56, 0.79***	0.65	0.55, 0.77***

Model 1: the association between obesity and an NCD was adjusted for age, sex, ethnicity, marital status, employment status, the number of years in education, and wealth index. Model 2: Model 1 with an additional adjustment for *an index of overall psychological well-being*. Index of overall psychological well-being was developed by re-standardising the average standardised scores of 10 psychological well-being related measures (depressive symptoms, life satisfaction, loneliness, positive affect, negative affect, purpose in life, anxiety, hopelessness, pessimism, and personal constraint). Comparisons between overweight vs. normal weight for each NCD are not presented.

*n* analytical sample size (excluding participants with the outcome before and at baseline and missing observations), *HR* hazard ratio, *CI* confidence interval.

*p < 0.05; **p < 0.01; ***p < 0.001.

The index of overall psychological well-being was associated with heart disease, stroke, arthritis, and memory-related disease, independently of obesity status in HRS (Table 6). These findings were largely consistent when individual psychological well-being related measures were examined (Table C2). In addition, stronger associations between obesity and psychological well-being related measures appeared when Class II & III obesity and normal weight were compared (Table C3), as opposed to all obesity vs. normal weight. Findings from mediation analyses in HRS were similar to ELSA. The associations between obesity and increased risk of some NCDs were not mediated by index of overall psychological well-being (Table 7) and individual psychological well-being related measures that satisfied pre-mediation conditions (Table C4).

**Table 6:** Longitudinal associations between an index of overall psychological well-being and the incidence of NCDs in HRS.

Outcomes	Index of overall psychological well-being measures				
	*n*	Model 1		Model 2	
		HR	95% CI	HR	95% CI
Hypertension	2773	1.06	0.98, 1.15	1.06	0.97, 1.15
Heart disease	7443	1.15	1.07, 1.23***	1.15	1.07, 1.23***
Stroke	9440	1.29	1.18, 1.41***	1.29	1.18, 1.41***
Diabetes	7594	1.08	0.99, 1.17	1.07	0.99, 1.16
Arthritis	3486	1.24	1.15, 1.34***	1.24	1.15, 1.34***
Cancer	8609	1.05	0.97, 1.13	1.04	0.97, 1.13
Memory-related disease	9462	1.41	1.32, 1.50***	1.41	1.32, 1.51***

Model 1: the association between obesity and an NCD was adjusted for age, sex, ethnicity, marital status, employment status, the number of years in education, and wealth index. Model 2: Model 1 with an additional adjustment for *obesity status*. Index of overall psychological well-being was developed by re-standardising the average standardised scores of 10 psychological well-being related measures (depressive symptoms, life satisfaction, loneliness, positive affect, negative affect, purpose in life, anxiety, hopelessness, pessimism, and personal constraint). Comparisons between overweight vs. normal weight for each NCD are not presented.

*n* analytical sample size (excluding participants with the outcome before and at baseline and missing observations), *HR* hazard ratio, *CI* confidence interval.

*p < 0.05; **p < 0.01; ***p < 0.001.

**Table 7:** Mediation by an index of overall psychological well-being in the longitudinal associations between obesity and the incidence of NCDs in HRS.

Outcomes	Mediation by an index of overall psychological well-being measures					
	Obesity vs. normal weight			Obesity class II & III vs. normal weight		
	*n*	Estimate	95% CI	*n*	Estimate	95% CI
Hypertension	1659	0.001	−0.010, 0.012	1115	−0.077	−0.244, 0.090
Heart disease	4742	0.003	−0.018, 0.023	2834	0.002	−0.019, 0.023
Stroke	NA			3611	0.065	−0.048, 0.179
Diabetes	4687	−0.001	−0.002, 0.001	2911	0.004	−0.004, 0.012
Arthritis	2135	−0.039	−0.093, 0.015	1309	−0.021	−0.079, 0.047

All the mediation analyses were adjusted for age, sex, ethnicity, marital status, employment status, the number of years in education, and wealth index. Index of overall psychological well-being was developed by re-standardising the average standardised scores of 10 psychological well-being related measures (depressive symptoms, life satisfaction, loneliness, positive affect, negative affect, purpose in life, anxiety, hopelessness, pessimism, and personal constraint). As only a binary or continuous independent variable is allowed, causal mediation analysis excluded participants with overweight for the comparison between obesity vs. normal weight, and participants with overweight and Class I obesity for the comparison between Class II & III obesity vs. normal weight, resulting in a smaller analytical sample size compared to previous regression analyses that allowed an ordinal independent variable to be fitted.

*n* analytical sample size (excluding participants with the outcome before and at baseline and missing observations), *Estimate* the overall proportion due to mediation or the proportion mediated, *CI* confidence interval, *NA* not applicable (e.g., no significant risk differences in developing stroke between participants with obesity vs. normal weight at *p* < 0.05—see Table 4).

*p < 0.05; **p < 0.01; ***p < 0.001.

When the outcome was assessed as the cumulative number of NCDs, obesity (IRR = 1.13; 95% CI = 1.11, 1.16) and psychological well-being related measures (e.g., index of overall psychological well-being measures, life satisfaction, anxiety) were associated with the cumulative number of NCDs (Tables C5 and C6, respectively). Psychological well-being related measures did not mediate the association between obesity and the number of NCDs (Table C7). Furthermore, we did not assess mediation by change in BMI between waves on the association between baseline psychological well-being related measures and NCDs as preconditions were not satisfied (see Tables C8 and C9). In HRS, no evidence for the interaction between obesity and index of overall psychological well-being was observed in predicting NCDs (Table C10).

## Discussion

In UK (ELSA) and US (HRS) samples of older adults, obesity (vs. normal weight) was consistently associated with an increased risk of developing a range of NCDs (hypertension, heart disease, diabetes, and arthritis). We examined the potential role of a wide range of psychological well-being related measures in explaining these obesity-NCD risks. Statistical evidence indicated that a large number of the psychological measures examined were inter-related and corresponded to an overall factor of psychological well-being. This index of overall psychological well-being and a number of the psychological well-being related measures when treated individually were associated with an increased risk of heart disease, stroke, arthritis, and memory-related disease, independently of obesity status. However, there was no evidence that the reason obesity was associated with increased NCD risk was in part explained by psychological well-being related measures (as an overall index or individually) associated with obesity in both ELSA and HRS. Findings were consistent across additional analyses, including when the cumulative number of NCDs developed were considered as the outcome, and when NCDs derived from linked hospital admissions data were used (ELSA). Additionally, we found no evidence that BMI change explained why some individual psychological well-being related measures increased risk of NCD incidence. There was weak evidence for psychological well-being related measures moderating the associations between obesity and NCDs. In summary, obesity and psychological well-being may independently and additively increase risk of developing NCDs.

The psychological burden of obesity due to exposure to chronic stressors (e.g., weight stigma) has been proposed to have future health impacts [17, 18]. In line with this, a previous study reported that having excess visceral fat is associated with weight stigma across BMI categories, particularly in women [46]. In our analyses, the associations between obesity and psychological well-being related measures tended to be more salient in participants with obesity Class II & III vs. normal weight. This may be because participants with obesity Class II & III experience more stigmatisation [19]. Impaired psychological well-being, as evidenced by depressive symptoms for example, may lead to dysregulation of multiple physiological functioning [17, 31, 47], leading to the proposal that obesity related increased risk of disease may be explained by (mediated) the psychosocial burden of obesity [48, 49]. Contrary to this proposition, our findings indicated that psychological well-being related measures did not explain why obesity was associated with risk of disease incidence. This aligns with our previous findings that a range of psychological well-being related measures did not convincingly explain worsening physiological health in the UK and the US older adults with obesity [10], with the possible exception of experiencing weight discrimination contributing to a modest amount of obesity-related worsening of physiological health over time. In the present analyses, we were unable to examine reported experiencing of weight discrimination as a potential mediator of obesity related NCD incidence, because of a lack of weight discrimination measure at baseline and concern that the timing of measurement in waves may produce results prone to reverse causality (e.g., weight gain from baseline onwards leading to perceived weight discrimination and NCD incidence). Given the likely damaging effects of experiencing discrimination [50] this remains an important variable to consider in the future.

Based on the present results, the prospective association between obesity and NCDs in the present study may be largely driven by a direct effect of adiposity. Visceral adipose tissue is highly metabolically active and linked to constant free fatty acids (FFA) release that leads to the development of metabolic syndrome, including systemic inflammation, hyperinsulinemia, dyslipidaemia, and atherosclerosis [5, 6, 51]. Even though overall obesity is generally correlated with central or abdominal obesity (i.e., excessive concentration of visceral body fat); some individuals who fall within the obesity category may not have abdominal obesity [52]. Individuals with normal BMI but with excess visceral fat tend to have a more adverse metabolic profile than those with elevated BMI (≥25 kg/m^2^) but with low visceral fat [53]. Furthermore, an unhealthy metabolic profile has been found to be associated with worsening physical health, independently of obesity status. For example, dyslipidaemia (e.g., increasing total cholesterol, decreasing high-density lipoprotein (HDL)) is monotonically associated with increased risk of cardiovascular disease in all BMI groups [54].

It is important to note that null findings on mediation by psychological well-being related measures in this study may be also due to the complex association between obesity and psychological well-being related measures in older age participants. Evidence from a meta-analysis indicates that current obesity status may be a protective factor against depression in older adults [55]. Older adults may experience unintentional weight loss due to some health conditions that may be also associated with obesity (e.g., cancer, gastrointestinal disease, cognitive impairment) [56], and this obesity-related morbidity has been found to be related to reduced psychological well-being [57]. Examining current weight status alone in older age participants may fail to address the role of previous obesity status in initiating subsequent adverse physical consequences that contribute to weight loss and worse psychological outcomes [41].

Aside from obesity, other physical and social conditions also contribute to impaired psychological well-being in older adults, including chronic diseases, chronic pain and disability, changes in financial circumstances and social status [58]. Worsening physiological health may partly explain the impact of impaired psychological well-being on future NCDs. In line with this, metabolic syndrome is more common in individuals with depression [32]. A previous study also indicated that a range of psychological well-being related measures was associated with physiological health in the UK older adults independent of obesity [10]. In addition, some mechanisms through vascular disease and hippocampal atrophy have been proposed as pathways linking depression to memory-related disease (e.g., dementia) [59]. Moreover, psychological well-being related measures may also be indirectly associated with NCDs through promoting health risk behaviours, including unhealthy eating [33], physical inactivity [34], and smoking [60].

### Strengths and limitations

Our study is the first comprehensive assessment of the role of a wide range of psychological well-being related measures in explaining obesity-related NCD incidence. We used two large longitudinal datasets from the UK and the US to examine the generalisability of the findings. Limitations included use of self-reported NCDs in primary analyses for ELSA and HRS. However, the findings remained the same in analyses where linked hospital admission data were available to diagnose NCDs (ELSA). Nevertheless, NCDs derived from hospital admissions data may be under-reported as individuals with NCD conditions may be treated in primary care for these conditions, as opposed to being hospitalised. In addition, this study only included older age (≥50 years) and mostly White participants, and therefore, future studies will benefit from replicating the analyses in younger and more ethnically diverse participants. Like in the majority of studies examining the obesity-related disease burden we investigated how baseline obesity status predicted future disease risk. However, future work examining both current and history of obesity will be important to understand the impact of obesity on psychological well-being related measures and subsequent health outcomes developed in older adults. Psychological well-being related measures were examined at a single time-point in this study, and therefore modelling the potential dynamic nature of the association between body weight, psychological well-being, and the onset of NCDs was not possible. Future work will benefit from understanding this potential dynamic association.

## Conclusion

The prospective association between obesity and risk of developing NCDs were not explained by psychological well-being related measures. Obesity and psychological well-being may independently and additively increase risk of developing NCDs.

### Supplementary information

Supplementary materials

## Data Availability

Data are available online (ELSA: 10.5255/UKDA-SN-5050-25; HRS: https://hrsdata.isr.umich.edu/data-products/rand) with the permission of the data custodians.
