# Supplementary material for: Obesity, psychological well-being related measures, and risk of seven non-communicable diseases: evidence from longitudinal studies of UK and US older adults
Source: Int J Obes (Lond). 2024 Jun 1;48(9):1283–91. doi: 10.1038/s41366-024-01551-1 (PMC11347379; doi:10.1038/s41366-024-01551-1)
Supplement: Supplementary file 1 — Supplementary materials [file 41366_2024_1551_MOESM1_ESM.pdf]

Supplementary Materials

Supplementary information

Exposure: Obesity

In ELSA, a nurse assessed participants' weight without shoes and in light clothing using Tanita THD-305 portable electronic scales to the nearest 0.1 kg and height without shoes using a portable stadiometer to the nearest millimetre [1]. In HRS, as part of EFTF, a trained interviewer used the Healthometer 830KL scale to measure participants' weight in light clothing without shoes to the nearest half pound. A tape measure was then used to measure standing height without shoes to the nearest quarter inch [2]. In both studies, BMI was calculated as weight in kilogram (kg) divided by height in metres squared (m^2).

Candidate mediators: Psychological well-being related measures

1. Depressive symptoms

   An 8-item version of the Centre for Epidemiology Depression Scale (CED-S) was used to quantify depressive symptoms [3]. Participants were asked whether they felt negative emotions during the past week (e.g., "*did you feel depressed?*", *"did you feel everything you did was an effort"*) with "*yes*" and "*no*" options of answers. All the responses were added together to create a total score ranging from 0 to 8. A higher score indicates greater depressive symptoms.

2. Enjoyment of life

   Enjoyment of life was defined following previous studies [4, 5]. This was assessed using four questions (e.g., *"I enjoy the things that I do," "I enjoy being in the company of others"*) from the Control, Autonomy, Self-Realization and Pleasure (CASP-19) CASP-19 quality of life questionnaire [6]. Responses on a 4-point Likert scale from 0= "*often*" to 3= "*never*" were reverse-coded and summed. An enjoyment of life index ranged from 0 to 12 with a higher score indicating greater enjoyment of life.

3. Eudemonic well-being

   Following a previous approach [7], eudemonic well-being was determined by adding together the responses of the remaining 15 items from CASP-19 that were not used to define enjoyment of life (e.g., *"How often feels what happens to them is out of their control", "How often feels left out of things"*). The total score ranged from 0 to 45 with a higher score indicating a greater level of eudemonic well-being.

4. Life satisfaction

   Life satisfaction was assessed using the Satisfaction with Life Scale (SWLS) [8]. This scale consisted of five 7-point Likert-scale items (e.g., "*in most ways my life is close to my ideal*", *"the conditions of my life are excellent"*) (1= "*strongly disagree"* to 7= "*strongly agree")*. The summary score was determined by summing all responses. A higher score on a possible range of 1-to-35 for a total score indicated greater life satisfaction.

5. Loneliness

   Following a previous study [9], loneliness was measured using a three-item loneliness scale [10], adopted from the 20-item Revised UCLA loneliness scale [11]. The following questions were asked to the participants, *"How often do you feel: you lack companionship?", "isolated from others?", "left out?")* with three options of answers, 1= "*hardly ever or never*," 2= "*some of the time*," and 3= "*often*". The summary score on a possible range of 1-to-9 was generated by summing responses of all the items with a higher score indicating a higher loneliness.

6. Social support

   Participants' reports on positive experiences of social support for different types of relationships (spouse or partner, children, other immediate family members, and friends) were used to define social support. Three questions were asked to participants (e.g., "*How much do they really understand the way you feel about things?"*, "*How much can you rely on them if you have a serious problem?*") for each relationship with four options of answers available (1= "*a lot*" to 4= "*not at all*") (e.g., as in Khondoker, Rafnsson et al [12]). Responses to each question were reverse-coded to make a higher value indicate more positive experience and then averaged for each relationship. The final summary score which represents overall social support was generated by re-averaging the social support scores from all the reported relationships.

7. Social strain

   Social strain was defined in the same manner as social support. Social strain indicated negative social experiences received from different relationships as were assessed for social support. Three items on a 4-point-Likert scale were used to evaluate social strain (e.g., "*How much do they criticise you?*", "*How much do they let you down when you are counting on them?*") from each relationship (1= "*a lot*" to 4= "*not at all*") (e.g., as in Khondoker, Rafnsson et al [12]). Responses were reverse-coded to indicate more negative experiences for a greater value and then averaged for each relationship. An overall social strain score ranging from 1 to 4 was calculated by re-averaging the average social strain scores from all reported relationships.

8. Positive affect

   Thirteen items from the Positive and Negative Affect scale (PANAS-X) were used to evaluate positive affect [13]. Participants were asked how they felt in the past 30 days (e.g., "*During the last 30 days, to what degree did you feel: determined?", "enthusiastic?"*) with five options of responses available (1= "*not at all*"" to 5= "*very much*"). A summary score was generated by adding together all responses. Greater positive affect was indicated by a higher score on a scale from 1 to 65.

9. Negative affect

   Negative affect was assessed using the remaining 12 items from PANAS-X [13]. Participants were asked the extent to which they felt negative emotions in the last 30 days (e.g., "*During the last 30 days, to what degree did you feel: afraid?", "upset?"*). As with positive affect, participants' answers on a 5-point-Likert scale (1= "*not at all*"" to 5= "*very much*") were summed. A total score ranged from 1 to 60 with a higher score indicating greater negative affect.

10. Purpose in life

    A 7-item domain of purpose in life from the Ryff Measures of Psychological Wellbeing [14, 15] was used to evaluate purpose in life. Participants' responses to 6-point-Likert-scale items (e.g., "*I enjoy making plans for the future and working to make them a reality.*", "*I am an active person in carrying out the plans I set for myself.*") (1= "*strongly disagree*" to 6= "*strongly agree*") were added together to create a total score ranging from 1 to 42. A higher score indicated greater purpose in life.

11. Anxiety

    Five items from Beck Anxiety Inventory (BAI) were used to quantify anxiety [16]. Participants responded to what degree they felt the following situations in the past week (e.g., "*I had fear of the worst happening*", "*I was nervous*") with four possible responses (1= *"never"* to 4= *"most of the time"*). A total score was generated by adding together all responses. Greater anxiety was indicated by a higher score on a scale from 1 to 20.

12. Hopelessness

    Two items adapted from Beck, Weissman et al [17] and two from Everson, Kaplan et al [18] (e.g., "*I feel it is impossible for me to reach the goals that I would like to strive for.*", "*I don't expect to get what I really want.*") on a 6-point Likert scale (1= "*strongly disagree*" to 6= "*strongly agree*") were used to evaluate hopelessness. All the responses were summed, resulting in a summary score ranging from 1 to 24 with a higher score indicating greater hopelessness.

13. Optimism

    Optimism was measured using three items (e.g., "*I'm always optimistic about my future.*", "*In uncertain times, I usually expect the best.*") on a 6-point Likert scale (1= "*strongly disagree*" to 6= "*strongly agree*") from the Life Orientation Test-Revised (LOT-R) [19]. A summary score ranging from 1 to 18 was computed by totalling the items' responses. A higher score indicated greater optimism.

14. Pessimism

    As with optimism, the remaining three items from the LOT-R (e.g., "*If something can go wrong for me it will.*", "*I hardly ever expect things to go my way.*") on a 6-point Likert scale (1= "*strongly disagree*" to 6= "*strongly agree*") were used to assess pessimism [19] A total score ranging from 1 to 18 was created by adding all the responses. A higher score indicated greater pessimism.

15. Cynical hostility

    The Cook-Medley Hostility Inventory was used to evaluate cynical hostility. Participants' responses to five 6-point-Likert-scale questions (e.g., "*Most people dislike putting themselves out to help other people.*", "*Most people will use somewhat unfair means to gain profit or an advantage rather than lose it.*") (1= "*strongly disagree*" to 6= "*strongly agree*") were added to create a total score [20, 21]. A higher score on a possible range of 1-to-30 for a total score indicated greater cynical hostility.

16. Personal constraints

    Participants were asked five items regarding perceptions of constraints over their life circumstances (e.g., "*I often feel helpless in dealing with the problems of life.*", "*Other people determine most of what I can and cannot do.*"). Their answers on a 6-point Likert scale (1= "*strongly disagree*" to 6=

"*strongly agree"*) were added to generate a total score [22, 23]. A higher level of perceived constraints is indicated by a higher total score on a scale from 1 to 30.

17. Mastery

    Five items on a 6-point Likert scale (1= "*strongly disagree"* to 6= "*strongly agree"*) regarding beliefs to perform specific behaviours (e.g., "*I can do just about anything I really set my mind to.*", "*When I really want to do something, I usually find a way to succeed at it.*") were used to assess the level of perceived mastery [22, 23]. Participants' responses to all the items were totalled to create a summary score ranging from 1 to 30. A higher score indicates greater perceived mastery.

Main analyses

Causal mediation analysis using the 'med4way' command in STATA version 17 [24] was used to examine psychological well-being related measures as a potential explanation of the association between obesity and risk of developing NCDs. This mediation analysis allows a survival outcome with binary or continuous independent and mediator variables. Separate single mediation models were developed to examine mediation by an index of overall psychological well-being and some psychological well-being related measures in the association between obesity and an NCD, controlling for sociodemographic covariates. Participants who developed an NCD before and up to baseline were excluded from mediation analysis for that NCD. This causal mediation analysis decomposed the total effect into four components: (1) controlled direct effect ("not explained by either the mediator or exposure-mediator interaction; explained only by the exposure"), (2) reference interaction ("explained only by the interaction between the exposure and the mediator"), (3) mediated interaction ("explained by both interaction and mediation or mediated interaction"), and (4) pure indirect effect ("explained only by mediation") [24, 25]. Both proportions due to mediated interaction and pure indirect effect together constitute the overall proportion due to mediation or the proportion mediated [25].

Mediation analysis using med4way fitted two regression models of the exposure on the mediator and the exposure on the outcome adjusted for and in interaction with the potential mediator. We selected linear regression for a model of obesity on psychological well-being related measure. To estimate the effect of obesity on NCDs, an accelerated failure time (AFT) model with a Weibull distribution was used (e.g., as in Karlsson, Zhan et al [26]) because Cox regression might provide biased estimates in mediation analyses of non-rare outcomes [27, 28]. As psychological well-being related measures were on a continuous scale, mediation at mean levels of the mediator was tested (e.g., as in Karlsson, Zhan et al [26]).

Across ELSA and HRS, we found that some sociodemographic characteristics (e.g., being older, male, non-White, not currently married, in lower household wealth quintiles) were associated with being excluded from the analysis due to missing observations. To address potential selection bias, we used inverse probability weighting approach [29, 30]. First, we included sociodemographic covariates in a logistic regression model to estimate the probability of being retained at follow-up or included in the analytic sample. New sample weights were then calculated as the inverse probability of being retained and combined with baseline weights. We applied these combined weights in all the analyses to

compensate for the differences in participants' i) non-response characteristics at baseline and ii) characteristics associated with being retained at follow-up.

Additional analyses

We examined whether obesity at baseline was associated with the cumulative number of NCDs (min=0, max=7) reported across the follow-up period, to assess overall disease burden. We assessed this association by controlling the number of NCDs at baseline, and therefore, participants with one or more NCDs at baseline were not excluded to reduce further sample loss. As the outcome was a count variable, Poisson regression was used for this analysis. If we found evidence of this association, we would further examine mediation by psychological well-being related measures using med4way with a count outcome. Findings were presented as incidence-rate ratio (IRR) along with 95% confidence intervals (CI) and p-values.

We conducted robustness comparative analysis for ELSA by linking ELSA data with the Admitted Patient Care (APC) data from the National Health Service (NHS) Hospital Episode Statistics (HES) (e.g., as in Bu, Zaninotto et al [31]). This is because the list of NCDs available in ELSA is based on self-report and not exhaustive. Therefore, APC data can provide more accurate information on the diagnosis of some NCDs than self-reports in ELSA. We attempted to replicate the main analysis using information on NCDs reported in APC data. For the analysis predicting an NCD, we excluded participants with that condition at baseline, either self-reported in ELSA or identified from a diagnosis reported in APC data (e.g., as in Bu, Zaninotto et al [31]). Cox-proportional hazard regression models were then used to examine the association between obesity and each NCD from APC data, and causal mediation analysis was used to examine mediation by psychological well-being related measures.

We also conducted additional analyses that were not pre-registered. Psychological well-being related measures (e.g., depressive symptoms) have been shown to be associated with weight changes [32] and this may increase risk of developing NCDs [33]. Therefore, we examined the extent to which weight changes between waves (between Waves 4 and 6 in ELSA; and between Waves 9–10 and 11–12 in HRS) explained the prospective associations between psychological well-being related measures at baseline and NCDs (as opposed that psychological well-being related measures explained the associations between obesity and NCDs). For these analyses, the follow-up period for each NCD began from Wave 6 for ELSA and Waves 11–12 for HRS.

Supplementary tables

Table A1. Obesity-related NCDs and supporting studies

Obesity-related NCDs	Associations with obesity	Associations with psychological well-being related measures
Hypertension	Aronow [34], Kim, Kim et al [35]	Shay, MacKinnon et al [36], Pan, Cai et al [37]
Heart diseases	Kim, Kim et al [35], Riaz, Khan et al [38]	Van der Kooy, van Hout et al [39], Gan, Gong et al [40]
Stroke	Kim, Kim et al [35]	Pan, Sun et al [41], Pérez-Piñar, Ayerbe et al [42]
Diabetes	Riaz, Khan et al [38]	Roy and Lloyd [43], Kan, Silva et al [44]
Arthritis	Zheng and Chen [45], Crowson, Matteson et al [46]	Fakra and Marotte [47]
Cancer	Dobbins, Decorby et al [48]	Wang, Li et al [49]
Dementia or memory-related diseases	Park, Ko et al [50], Beydoun, Beydoun et al [51]	Cherbuin, Kim et al [52], Stafford, Chung et al [53]

Table A2. Descriptions of obesity-related NCDs in ELSA and HRS

Obesity-related NCDs	English Longitudinal Study of Ageing (ELSA)	Health and Retirement Study (HRS)
Hypertension	***Self-reported diagnosis***<br>"Has a doctor ever told that (or had) any of the conditions on this card?" "High blood pressure or hypertension"<br>**- OR -**<br>***Biomarker assessment***<br>Blood pressure ≥ 140/90 mmHg [54]	***Self-reported diagnosis***<br>"Has a doctor ever told you that you have high blood pressure or hypertension?"<br>**- OR -**<br>***Biomarker assessment***<br>Blood pressure ≥ 140/90 mmHg [54]
Heart diseases	***Self-reported diagnosis***<br>"Has a doctor ever told that (or had) any of the conditions on this card?" "Angina", "A heart attack (including myocardial infarction or coronary, thrombosis)", "Congestive heart failure", "A heart murmur", "An abnormal heart rhythm", "Any other heart trouble"	***Self-reported diagnosis***<br>"Has a doctor ever told you that you had a heart attack, coronary heart disease, angina, congestive heart failure, or other heart problems?"
Stroke	***Self-reported diagnosis***<br>"Has a doctor ever told that (or had) any of the conditions on this card?" "A stroke (cerebral vascular disease)"	***Self-reported diagnosis***<br>"Has a doctor ever told you that you had a stroke?"
Diabetes	***Self-reported diagnosis***<br>"Has a doctor ever told that (or had) any of the conditions on this card?" "Diabetes or high blood sugar"<br>**– OR –**<br>***Biomarker assessment***<br>Fasting plasma glucose ≥ 7.0 mmol/L (126 mg/dL) [55] **– OR –** HbA1c ≥ 48 mmol/mol (6.5%) [56]	***Self-reported diagnosis***<br>"Has a doctor ever told you that you have diabetes or high blood sugar?"<br>**– OR –**<br>***Biomarker assessment***<br>HbA1c ≥ 48 mmol/mol (6.5%) [56]
Arthritis	***Self-reported diagnosis***<br>"Has a doctor ever told that (or had) any of the conditions on this card?" "Arthritis"	***Self-reported diagnosis***<br>"Have you ever had, or has a doctor ever told you that you have arthritis or rheumatism?"
Cancer	***Self-reported diagnosis***<br>"Has a doctor ever told that (or had) any of the conditions on this card?" "Cancer or a malignant tumour"	***Self-reported diagnosis***<br>"Has a doctor ever told you that you have cancer or a malignant tumor, excluding minor skin cancer?"
Dementia or memory-related diseases	***Self-reported diagnosis***<br>"Has a doctor ever told that (or had) any of the conditions on this card?" "Dementia, senility or any other serious memory impairment"<br>**– OR –**<br>***Interview***<br>The total scores from responses to 27-point modified Telephone Interview for Cognitive Status was used to classify participants into probable dementia (a score of 0–6) [57-59].	***Self-reported diagnosis***<br>"Has a doctor ever told you that you have a memory-related disease?"<br>**– OR –**<br>***Interview***<br>The total scores from responses to 27-point modified Telephone Interview for Cognitive Status was used to classify participants into probable dementia (a score of 0–6) [57-59].

	**– OR –**<br>The average scores from responses to the short-form Informant Questionnaire on Cognitive Decline in the Elderly (IQCODE) questionnaire (16 questions) was used to classify participants into probable dementia (an average score of ≥ 3.5 on a scale from 1 to 5) [12, 60].	**– OR –**<br>The average scores from responses to the short-form Informant Questionnaire on Cognitive Decline in the Elderly (IQCODE) questionnaire (16 questions) was used to classify participants into probable dementia (an average score of ≥ 3.5 on a scale from 1 to 5) [12, 60].

Compared to a pre-registered study protocol (https://doi.org/10.17605/OSF.IO/JRQAP), we also included an additional assessment of dementia using the short-form IQCODE questionnaire in this study.

Table A3. Psychological well-being related measures available in ELSA and HRS

Psychological well-being related measures	ELSA	HRS
Depressive symptoms	Wave 4	Waves 9 & 10
Enjoyment of life	Wave 4	-
Eudemonic wellbeing	Wave 4	-
Life satisfaction	Wave 4	Waves 9 & 10
Loneliness	Wave 4	Waves 9 & 10
Social support	Wave 4	Waves 9 & 10
Social strain	Wave 4	Waves 9 & 10
Positive affect	-	Waves 9 & 10
Negative affect	-	Waves 9 & 10
Purpose in life	-	Waves 9 & 10
Anxiety	-	Waves 9 & 10
Hopelessness	-	Waves 9 & 10
Optimism	-	Waves 9 & 10
Pessimism	-	Waves 9 & 10
Cynical hostility	-	Waves 9 & 10
Personal constraint	-	Waves 9 & 10
Mastery	-	Waves 9 & 10

ELSA: Wave 4 (2008-2009; baseline); HRS: Waves 9 & 10 (2008-2010; baseline)

Findings from the English Longitudinal Study of Ageing (ELSA)

Table B1. Longitudinal associations between Class II & III obesity (vs. normal weight) and the incidence of NCDs in ELSA

**Outcomes**	**Class II & III obesity vs. normal weight**				
	**n**	**Model 1**		**Model 2**	
		**HR**	**95% CI**	**HR**	**95% CI**
Hypertension	2,699	1.77	1.35, 2.33***	1.72	1.31, 2.27***
Heart disease	5,082	1.91	1.50, 2.42***	1.88	1.48, 2.39***
Stroke	6,181	1.16	0.74, 1.81	1.13	0.73, 1.76
Diabetes	5,629	6.27	4.21, 9.35***	6.25	4.19, 9.32***
Arthritis	3,929	1.87	1.41, 2.48***	1.84	1.39, 2.44***
Cancer	5,870	1.05	0.76, 1.45	1.04	0.75, 1.44
Memory-related disease	6,376	1.03	0.68, 1.57	0.96	0.62, 1.48

**p<0.05; **p<0.01; ***p<0.001*

n=analytical sample size (excluding participants with the outcome before and at baseline and missing observations); HR=hazard ratio; CI=confidence interval

Model 1: The association between obesity and an NCD was adjusted for age, sex, ethnicity, marital status, paid employment status, completion of degree qualification level, and wealth index.

Model 2: Model 1 with an additional adjustment for an index of overall psychological well-being.

Index of overall psychological well-being was developed by re-standardizing the average standardized scores of five psychological well-being related measures (depressive symptoms, enjoyment of life, eudemonic well-being, life satisfaction, and loneliness).

Comparisons between overweight vs. normal weight and between Class I obesity vs. normal weight are not presented.

Table B2. Longitudinal associations between psychological well-being related measures and the incidence of NCDs in ELSA

	n	Model 1		Model 2	
		HR	95% CI	HR	95% CI
**Outcome: Hypertension**					
Depressive symptoms	2,697	1.03	0.95, 1.12	1.02	0.94, 1.11
Enjoyment of life	2,699	0.87	0.81, 0.93***	0.87	0.81, 0.94***
Eudemonic well-being	2,697	0.90	0.83, 0.98*	0.91	0.84, 0.99*
Life satisfaction	2,680	0.93	0.86, 1.00	0.93	0.86, 1.00
Loneliness	2,694	1.09	1.00, 1.18	1.08	1.00, 1.17
Social support	2,698	0.97	0.90, 1.05	0.98	0.91, 1.05
Social strain	2,697	1.06	0.98, 1.14	1.05	0.97, 1.13
**Outcome: Heart disease**					
Depressive symptoms	5,080	1.12	1.04, 1.20**	1.11	1.03, 1.20**
Enjoyment of life	5,076	0.89	0.84, 0.95**	0.90	0.84, 0.96**
Eudemonic well-being	5,073	0.88	0.81, 0.95**	0.88	0.82, 0.95**
Life satisfaction	5,035	0.89	0.83, 0.96**	0.89	0.83, 0.95**
Loneliness	5,071	1.11	1.04, 1.20**	1.11	1.04, 1.19**
Social support	5,077	0.94	0.88, 1.01	0.94	0.88, 1.01
Social strain	5,075	1.05	0.98, 1.13	1.05	0.97, 1.12
**Outcome: Stroke**					
Depressive symptoms	6,178	1.11	0.98, 1.24	1.10	0.97, 1.24
Enjoyment of life	6,173	0.87	0.78, 0.98*	0.88	0.79, 0.98*
Eudemonic well-being	6,166	0.83	0.73, 0.94**	0.83	0.74, 0.95**
Life satisfaction	6,125	0.93	0.81, 1.06	0.93	0.81, 1.06
Loneliness	6,160	1.05	0.93, 1.19	1.04	0.92, 1.18
Social support	6,175	0.93	0.82, 1.06	0.94	0.83, 1.07
Social strain	6,173	0.97	0.85, 1.11	0.97	0.85, 1.11
**Outcome: Diabetes**					
Depressive symptoms	5,626	1.03	0.92, 1.15	1.01	0.90, 1.13
Enjoyment of life	5,622	0.93	0.84, 1.03	0.94	0.85, 1.04
Eudemonic well-being	5,616	0.95	0.85, 1.05	0.97	0.86, 1.08
Life satisfaction	5,579	1.00	0.90, 1.11	0.99	0.89, 1.10
Loneliness	5,610	1.05	0.94, 1.18	1.05	0.94, 1.17
Social support	5,622	0.87	0.78, 0.98*	0.88	0.79, 0.98*
Social strain	5,620	1.07	0.97, 1.20	1.06	0.96, 1.18
**Outcome: Arthritis**					
Depressive symptoms	3,928	1.17	1.08, 1.26***	1.17	1.08, 1.26***
Enjoyment of life	3,925	0.85	0.79, 0.90***	0.85	0.79, 0.91***
Eudemonic well-being	3,921	0.80	0.74, 0.86***	0.80	0.74, 0.86***
Life satisfaction	3,897	0.82	0.76, 0.88***	0.82	0.76, 0.88***
Loneliness	3,917	1.13	1.05, 1.22**	1.13	1.05, 1.22**
Social support	3,924	0.87	0.82, 0.94***	0.88	0.82, 0.94***
Social strain	3,922	1.10	1.02, 1.17**	1.09	1.01, 1.17*

	n	Model 1		Model 2	
		HR	95% CI	HR	95% CI
**Outcome: Cancer**					
Depressive symptoms	5,867	1.07	0.97, 1.18	1.06	0.96, 1.18
Enjoyment of life	5,862	0.96	0.87, 1.06	0.97	0.88, 1.07
Eudemonic well-being	5,856	0.99	0.90, 1.09	1.01	0.91, 1.11
Life satisfaction	5,815	0.94	0.85, 1.04	0.94	0.85, 1.04
Loneliness	5,850	1.03	0.93, 1.13	1.02	0.92, 1.13
Social support	5,863	0.98	0.90, 1.07	0.99	0.90, 1.07
Social strain	5,861	1.06	0.96, 1.17	1.05	0.96, 1.16
**Outcome: Memory-related disease**					
Depressive symptoms	6,374	1.27	1.15, 1.41***	1.27	1.15, 1.41***
Enjoyment of life	6,368	0.75	0.68, 0.82***	0.75	0.68, 0.82***
Eudemonic well-being	6,361	0.68	0.62, 0.76***	0.68	0.62, 0.76***
Life satisfaction	6,315	0.82	0.74, 0.92**	0.82	0.74, 0.92**
Loneliness	6,353	1.23	1.11, 1.36**	1.23	1.11, 1.36**
Social support	6,368	0.85	0.76, 0.94**	0.84	0.76, 0.94**
Social strain	6,366	1.18	1.06, 1.31**	1.18	1.06, 1.32**

**p<0.05; **p<0.01; ***p<0.001*
n=analytical sample size (excluding participants with the outcome before and at baseline and missing observations); HR=hazard ratio; CI=confidence interval
Model 1: The association between psychological well-being related measure and an NCD was adjusted for age, sex, ethnicity, marital status, paid employment status, completion of degree qualification level, and wealth index.
Model 2: Model 1 with an additional adjustment for obesity status.

Table B3. Cross-sectional associations between obesity and psychological well-being related measures in ELSA

Outcomes	n	Obesity vs. normal weight[a]		Class II & III obesity vs. normal weight[b]	
		β	95% CI	β	95% CI
Depressive symptoms	7,583	0.07	0.01, 0.13*	0.16	0.06, 0.26**
Enjoyment of life	6,866	-0.05	-0.12, 0.02	-0.10	-0.20, -0.01*
Eudemonic well-being	6,857	-0.08	-0.14, -0.01*	-0.18	-0.27, -0.09***
Life satisfaction	6,864	0.02	-0.05, 0.09	-0.02	-0.11, 0.07
Loneliness	6,856	0.03	-0.04, 0.09	0.17	0.07, 0.27**
Social support	6,937	-0.06	-0.13, 0.01	-0.12	-0.21, -0.02*
Social strain	6,931	0.16	0.09, 0.23***	0.24	0.14, 0.34***
Index of overall	6,874	0.05	-0.02, 0.11	0.16	0.06, 0.25**

**p<0.05; **p<0.01; ***p<0.001*

n=analytical sample size (excluding missing observations); β=regression coefficient; CI=confidence interval

[a]Comparisons between overweight vs. normal weight are not presented.

[b]Comparisons between overweight vs. normal weight and Class I obesity vs. normal weight are not presented.

All the associations were adjusted for age, sex, ethnicity, marital status, paid employment status, completion of degree qualification level, and wealth index.

Index of overall psychological well-being was developed by re-standardizing the average standardized scores of five psychological well-being related measures (depressive symptoms, enjoyment of life, eudemonic well-being, life satisfaction, and loneliness).

Table B4. Mediation by psychological well-being related measures in the longitudinal associations between obesity and the incidence of NCDs in ELSA

	Mediation by psychological well-being related measures		
	Class II & III obesity vs. normal weight		
	n	Estimate	95% CI
**Outcome: Heart disease**			
Depressive symptoms	1,884	0.031	-0.008, 0.071
Eudemonic well-being	1,882	0.049	-0.010, 0.109
Loneliness	1,882	0.026	-0.015, 0.067
**Outcome: Arthritis**			
Depressive symptoms	1,452	0.015	-0.021, 0.051
Eudemonic well-being	1,451	0.025	-0.022, 0.071
Loneliness	1,450	0.012	-0.028, 0.052
Social strain	1,448	0.004	-0.039, 0.046

**p<0.05; **p<0.01; ***p<0.001*

n=analytical sample size (excluding participants with the outcome before and at baseline and missing observations); Estimate=the overall proportion due to mediation or the proportion mediated; CI=confidence interval

All the mediation analyses were adjusted for age, sex, ethnicity, marital status, paid employment status, completion of degree qualification level, and wealth index.

As only a binary or continuous independent variable is allowed, causal mediation analysis excluded participants with overweight and Class I obesity for the comparison between Class II & III obesity vs. normal weight, resulting in a smaller analytical sample size compared to previous regression analyses that allowed an ordinal independent variable to be fitted.

See Tables 1 (main document), B1, B2, and B3 to determine whether preconditions for successful mediation were met.

Table B5. Longitudinal associations between obesity and the cumulative incidence of NCDs in ELSA

Outcomes	n	Model 1		Model 2	
		IRR	95% CI	IRR	95% CI
**Comparison: Obesity vs. normal weight[a]**					
A number of NCDs	6,425	1.35	1.26, 1.44***	1.35	1.26, 1.44***
**Comparison: Class II & III obesity vs. normal weight[b]**					
A number of NCDs	6,425	1.46	1.34, 1.59***	1.46	1.34, 1.59***

**p<0.05; **p<0.01; ***p<0.001*

n=analytical sample size (excluding missing observations); IRR=incidence-rate ratio; CI=confidence interval

[a]Comparisons between overweight vs. normal weight are not presented.

[b]Comparisons between overweight vs. normal weight and Class I obesity vs. normal weight are not presented.

Model 1: The association between psychological well-being related measure and a cumulative number of NCDs at follow-up was adjusted for <u>the number of NCDs at baseline,</u> age, sex, ethnicity, marital status, paid employment status, completion of degree qualification level, and wealth index.

Model 2: Model 1 with an additional adjustment for <u>an index of overall psychological well-being</u>.

Index of overall psychological well-being was developed by re-standardizing the average standardized scores of five psychological well-being related measures (depressive symptoms, enjoyment of life, eudemonic well-being, life satisfaction, and loneliness).

Table B6. Longitudinal associations between psychological well-being related measures and the cumulative incidence of NCDs in ELSA

	n	Model 1		Model 2	
		IRR	95% CI	IRR	95% CI
Depressive symptoms	6,422	1.01	0.99, 1.04	1.01	0.99, 1.04
Enjoyment of life	6,417	0.97	0.95, 0.99*	0.97	0.95, 1.00*
Eudemonic well-being	6,410	0.98	0.95, 1.00	0.98	0.95, 1.00
Life satisfaction	6,362	0.98	0.95, 1.00	0.98	0.95, 1.00
Loneliness	6,402	1.02	1.00, 1.05	1.02	0.99, 1.04
Social support	6,416	0.97	0.95, 1.00*	0.98	0.95, 1.00
Social strain	6,414	1.02	0.99, 1.04	1.01	0.99, 1.04
Index of overall psychological well-being	6,425	1.03	1.00, 1.06*	1.03	1.00, 1.06*

**p<0.05; **p<0.01; ***p<0.001*

n=analytical sample size (excluding missing observations); IRR=incidence-rate ratio; CI=confidence interval

Model 1: The association between psychological well-being related measure and a cumulative number of NCDs at follow-up was adjusted for <u>the number of NCDs at baseline,</u> age, sex, ethnicity, marital status, paid employment status, completion of degree qualification level, and wealth index.

Model 2: Model 1 with an additional adjustment for <u>obesity status</u>.

Index of overall psychological well-being was developed by re-standardizing the average standardized scores of five psychological well-being related measures (depressive symptoms, enjoyment of life, eudemonic well-being, life satisfaction, and loneliness).

Table B7. Longitudinal associations between psychological well-being related measures and changes in BMI (between Waves 4 and 6) in ELSA

Independent variables	BMI changes		
	**n**	**β**	**95% CI**
Depressive symptoms	5,668	-0.01	-0.09, 0.07
Enjoyment of life	5,221	-0.04	-0.11, 0.03
Eudemonic well-being	5,218	-0.03	-0.10, 0.04
Life satisfaction	5,223	-0.01	-0.08, 0.06
Loneliness	5,217	0.03	-0.05, 0.11
Social support	5,273	-0.01	-0.08, 0.06
Social strain	5,268	0.05	-0.02, 0.12
Index of overall psychological well-being	5,226	0.03	-0.05, 0.11

**p<0.05; **p<0.01; ***p<0.001*

n=analytical sample size (excluding missing observations); β=regression coefficient; CI=confidence interval

All the associations were adjusted for age, sex, ethnicity, marital status, paid employment status, completion of degree qualification level, and wealth index.

Index of overall psychological well-being was developed by re-standardizing the average standardized scores of five psychological well-being related measures (depressive symptoms, enjoyment of life, eudemonic well-being, life satisfaction, and loneliness).

Table B8. Longitudinal associations between changes in BMI (between Waves 4 and 6) and the incidence of NCDs in ELSA

**Outcomes**	**BMI changes**		
	**n**	**HR**	**95% CI**
Hypertension	1,913	1.05	0.95, 1.16
Heart disease	3,896	0.98	0.93, 1.04
Stroke	4,991	1.03	0.94, 1.13
Diabetes	4,678	1.06	0.96, 1.18
Arthritis	2,875	1.01	0.93, 1.10
Cancer	4,620	1.01	0.92, 1.10
Memory-related disease	5,176	0.95	0.89, 1.02

**p<0.05; **p<0.01; ***p<0.001*

n=analytical sample size (excluding participants with the outcome before and up to Wave 6 and missing observations); HR=hazard ratio; CI=confidence interval

All the associations were adjusted for age, sex, ethnicity, marital status, paid employment status, completion of degree qualification level, and wealth index.

Table B9. Longitudinal associations between obesity (vs. normal weight) and the incidence of NCDs reported from healthcare administrative data in ELSA

**Outcomes**	**Obesity vs. normal weight**				
	**n**	**Model 1**		**Model 2**	
		**HR**	**95% CI**	**HR**	**95% CI**
Hypertension	2,413	2.23	1.63, 3.04***	2.25	1.65, 3.06***
Heart disease	4,709	1.27	0.95, 1.71	1.28	0.95, 1.72
Stroke	5,839	1.13	0.74, 1.71	1.13	0.74, 1.71
Diabetes	5,291	3.80	2.38, 6.09***	3.81	2.38, 6.11***
Arthritis	3,696	1.56	0.45, 5.46	1.55	0.44, 5.38
Cancer	5,070	1.10	0.92, 1.31	1.10	0.92, 1.31
Memory-related disease	6,051	1.04	0.73, 1.50	1.04	0.72, 1.50

**p<0.05; **p<0.01; ***p<0.001*

n=analytical sample size (excluding participants with the outcome before and at baseline and missing observations); HR=hazard ratio; CI=confidence interval

Model 1: The association between obesity and an NCD was adjusted for age, sex, ethnicity, marital status, paid employment status, completion of degree qualification level, and wealth index.

Model 2: Model 1 with an additional adjustment for an index of overall psychological well-being.

Index of overall psychological well-being was developed by re-standardizing the average standardized scores of five psychological well-being related measures (depressive symptoms, enjoyment of life, eudemonic well-being, life satisfaction, and loneliness).

Comparisons between overweight vs. normal weight for each NCD are not presented.

Table B10. Longitudinal associations between Class II & III obesity (vs. normal weight) and the incidence of NCDs reported from healthcare administrative data in ELSA

**Outcomes**	**Class II & III obesity vs. normal weight**				
	**n**	**Model 1**		**Model 2**	
		**HR**	**95% CI**	**HR**	**95% CI**
Hypertension	2,413	4.01	2.76, 5.83***	3.96	2.71, 5.78***
Heart disease	4,709	1.70	1.17, 2.47**	1.68	1.15, 2.45**
Stroke	5,839	1.55	0.93, 2.59	1.55	0.92, 2.60
Diabetes	5,291	5.30	3.12, 9.01***	5.24	3.08, 8.93***
Arthritis	3,696	0.51	0.05, 5.15	0.49	0.05, 4.89
Cancer	5,070	0.93	0.73, 1.19	0.93	0.73, 1.20
Memory-related disease	6,051	1.19	0.73, 1.96	1.15	0.70, 1.88

**p<0.05; **p<0.01; ***p<0.001*
n=analytical sample size (excluding participants with the outcome before and at baseline and missing observations); HR=hazard ratio; CI=confidence interval
Model 1: The association between obesity and an NCD was adjusted for age, sex, ethnicity, marital status, paid employment status, completion of degree qualification level, and wealth index.
Model 2: Model 1 with an additional adjustment for an index of overall psychological well-being.
Index of overall psychological well-being was developed by re-standardizing the average standardized scores of five psychological well-being related measures (depressive symptoms, enjoyment of life, eudemonic well-being, life satisfaction, and loneliness).
Comparisons between overweight vs. normal weight and between Class I obesity vs. normal weight are not presented.

Table B11. Longitudinal associations between an index of overall psychological well-being and the incidence of NCDs reported from healthcare administrative data in ELSA

**Outcomes**	**Index of overall psychological well-being**				
	**n**	**Model 1**		**Model 2**	
		**HR**	**95% CI**	**HR**	**95% CI**
Hypertension	2,413	1.22	1.09, 1.37**	1.23	1.09, 1.38**
Heart disease	4,709	1.19	1.07, 1.33**	1.19	1.07, 1.32**
Stroke	5,839	1.02	0.88, 1.18	1.01	0.87, 1.17
Diabetes	5,291	1.17	1.01, 1.35*	1.14	0.99, 1.33
Arthritis	3,696	1.36	0.83, 2.24	1.36	0.82, 2.26
Cancer	5,070	1.00	0.92, 1.08	0.99	0.92, 1.08
Memory-related disease	6,051	1.21	1.04, 1.40*	1.21	1.04, 1.40*

**p<0.05; **p<0.01; ***p<0.001*
n=analytical sample size (excluding participants with the outcome before and at baseline and missing observations); HR=hazard ratio; CI=confidence interval
Model 1: The association between index of overall psychological well-being and an NCD was adjusted for age, sex, ethnicity, marital status, paid employment status, completion of degree qualification level, and wealth index.
Model 2: Model 1 with an additional adjustment for obesity status.
Index of overall psychological well-being was developed by re-standardizing the average standardized scores of five psychological well-being related measures (depressive symptoms, enjoyment of life, eudemonic well-being, life satisfaction, and loneliness).
Comparisons between overweight vs. normal weight for each NCD are not presented.

Table B12. Mediation by an index of overall psychological well-being in the longitudinal associations between obesity and the incidence of NCDs reported from healthcare administrative data in ELSA

**Outcomes**	**Mediation by an index of overall psychological well-being**					
	**Obesity vs. normal weight**			**Obesity class II & III vs. normal weight**		
	**n**	**Estimate**	**95% CI**	**n**	**Estimate**	**95% CI**
Hypertension	1,371	0.007	-0.010, 0.025	995	0.003	-0.017, 0.022
Heart disease	NA			1,748	0.003	-0.026, 0.033
Diabetes	2,997	0.001	-0.005, 0.007	1,918	0.003	-0.007, 0.014

**p<0.05; **p<0.01; ***p<0.001*

n=analytical sample size (excluding participants with the outcome before and at baseline and missing observations); Estimate=the overall proportion due to mediation or the proportion mediated; CI=confidence interval; ***NA=not applicable*** *(e.g., no significant risk differences in developing heart disease between participants with obesity vs. normal weight at p < 0.01 – see Table B9)*

All the mediation analyses were adjusted for age, sex, ethnicity, marital status, paid employment status, completion of degree qualification level, and wealth index.

Index of overall psychological well-being was developed by re-standardizing the average standardized scores of five psychological well-being related measures (depressive symptoms, enjoyment of life, eudemonic well-being, life satisfaction, and loneliness).

As only a binary or continuous independent variable is allowed, causal mediation analysis excluded participants with overweight for the comparison between obesity vs. normal weight, and participants with overweight and Class I obesity for the comparison between Class II & III obesity vs. normal weight, resulting in a smaller analytical sample size compared to previous regression analyses that allowed an ordinal independent variable to be fitted.

Table B13. Longitudinal associations between psychological well-being related measures and the incidence of NCDs reported from healthcare administrative data in ELSA

	n	Model 1		Model 2	
		HR	95% CI	HR	95% CI
**Outcome: Hypertension**					
Depressive symptoms	2,412	1.16	1.04, 1.29**	1.15	1.03, 1.29*
Enjoyment of life	2,413	0.83	0.75, 0.93**	0.83	0.75, 0.92**
Eudemonic well-being	2,413	0.76	0.67, 0.86***	0.76	0.67, 0.86***
Life satisfaction	2,397	0.94	0.83, 1.06	0.93	0.82, 1.05
Loneliness	2,410	1.08	0.96, 1.21	1.08	0.96, 1.22
Social support	2,412	0.91	0.82, 1.03	0.92	0.82, 1.03
Social strain	2,412	1.08	0.95, 1.22	1.07	0.94, 1.22
**Outcome: Heart disease**					
Depressive symptoms	4,707	1.19	1.07, 1.33***	1.19	1.07, 1.33**
Enjoyment of life	4,703	0.89	0.81, 0.99*	0.89	0.81, 0.99*
Eudemonic well-being	4,700	0.81	0.72, 0.90***	0.81	0.72, 0.91***
Life satisfaction	4,663	0.94	0.83, 1.05	0.93	0.83, 1.05
Loneliness	4,698	1.10	0.99, 1.23	1.10	0.99, 1.23
Social support	4,705	0.91	0.80, 1.02	0.90	0.80, 1.02
Social strain	4,704	1.08	0.96, 1.20	1.07	0.96, 1.19
**Outcome: Stroke**					
Depressive symptoms	5,836	1.04	0.90, 1.21	1.03	0.89, 1.20
Enjoyment of life	5,832	1.01	0.88, 1.15	1.02	0.89, 1.17
Eudemonic well-being	5,827	0.91	0.79, 1.05	0.92	0.80, 1.07
Life satisfaction	5,783	1.06	0.91, 1.24	1.06	0.91, 1.24
Loneliness	5,820	1.02	0.87, 1.19	1.01	0.87, 1.18
Social support	5,834	0.95	0.83, 1.10	0.95	0.83, 1.10
Social strain	5,833	1.09	0.93, 1.26	1.08	0.93, 1.26
**Outcome: Diabetes**					
Depressive symptoms	5,288	1.01	0.87, 1.17	0.98	0.85, 1.14
Enjoyment of life	5,285	0.87	0.76, 0.98*	0.89	0.78, 1.00
Eudemonic well-being	5,282	0.83	0.72, 0.96*	0.86	0.74, 1.00
Life satisfaction	5,243	0.91	0.77, 1.07	0.90	0.76, 1.06
Loneliness	5,275	1.17	1.01, 1.35*	1.16	1.00, 1.35*
Social support	5,285	0.83	0.71, 0.96*	0.84	0.73, 0.97*
Social strain	5,284	1.05	0.89, 1.23	1.04	0.90, 1.21
**Outcome: Arthritis**					
Depressive symptoms	3,694	1.21	0.77, 1.91	1.21	0.76, 1.91
Enjoyment of life	3,692	0.72	0.47, 1.08	0.72	0.47, 1.09
Eudemonic well-being	3,688	0.73	0.45, 1.19	0.73	0.44, 1.22
Life satisfaction	3,664	0.91	0.53, 1.55	0.90	0.53, 1.56
Loneliness	3,685	1.21	0.77, 1.91	1.21	0.77, 1.90
Social support	3,691	1.06	0.68, 1.66	1.08	0.69, 1.68
Social strain	3,690	1.12	0.74, 1.70	1.11	0.73, 1.68

	n	Model 1		Model 2	
		HR	95% CI	HR	95% CI
**Outcome: Cancer**					
Depressive symptoms	5,068	1.04	0.97, 1.12	1.04	0.96, 1.12
Enjoyment of life	5,063	0.99	0.92, 1.06	0.99	0.92, 1.07
Eudemonic well-being	5,060	1.01	0.94, 1.09	1.01	0.94, 1.09
Life satisfaction	5,023	1.00	0.93, 1.07	1.00	0.93, 1.08
Loneliness	5,054	0.96	0.89, 1.04	0.96	0.89, 1.03
Social support	5,065	1.03	0.97, 1.10	1.03	0.97, 1.10
Social strain	5,064	1.02	0.95, 1.08	1.01	0.95, 1.08
**Outcome: Memory-related disease**					
Depressive symptoms	6,049	1.08	0.93, 1.25	1.08	0.94, 1.25
Enjoyment of life	6,044	0.76	0.68, 0.84***	0.76	0.68, 0.84***
Eudemonic well-being	6,039	0.82	0.71, 0.96*	0.82	0.71, 0.95**
Life satisfaction	5,991	0.97	0.83, 1.13	0.97	0.83, 1.13
Loneliness	6,030	1.07	0.94, 1.22	1.07	0.94, 1.23
Social support	6,044	1.02	0.89, 1.17	1.02	0.90, 1.18
Social strain	6,043	0.94	0.81, 1.09	0.94	0.81, 1.08

**p<0.05; **p<0.01; ***p<0.001*

n=analytical sample size (excluding participants with the outcome before and at baseline and missing observations); HR=hazard ratio; CI=confidence interval

Model 1: The association between psychological well-being related measure and an NCD was adjusted for age, sex, ethnicity, marital status, paid employment status, completion of degree qualification level, and wealth index.

Model 2: Model 1 with an additional adjustment for obesity status.

Table B14. Mediation by psychological well-being related measures in the longitudinal associations between obesity and the incidence of NCDs reported from healthcare administrative data in ELSA

	**Mediation by psychological well-being related measures**		
	**Class II & III obesity vs. normal weight**		
	**n**	**Estimate**	**95% CI**
**Outcome: Hypertension**			
Depressive symptoms	1,085	0.010	-0.017, 0.037
Eudemonic well-being	995	-0.003	-0.027, 0.021
**Outcome: Heart disease**			
Depressive symptoms	1,907	0.001	-0.036, 0.039
Eudemonic well-being	1,745	0.020	-0.047, 0.086

**p<0.05; **p<0.01; ***p<0.001*
n=analytical sample size (excluding participants with the outcome before and at baseline and missing observations); Estimate=the overall proportion due to mediation or the proportion mediated; CI=confidence interval
All the mediation analyses were adjusted for age, sex, ethnicity, marital status, paid employment status, completion of degree qualification level, and wealth index.
As only a binary or continuous independent variable is allowed, causal mediation analysis excluded participants with overweight and Class I obesity for the comparison between Class II & III obesity vs. normal weight, resulting in a smaller analytical sample size compared to previous regression analyses that allowed an ordinal independent variable to be fitted.
See Tables B3, B9, B10, and B13 to determine whether preconditions for successful mediation were met.

Table B15. Longitudinal associations between obesity and the cumulative incidence of NCDs reported from healthcare administrative data in ELSA

**Outcomes**	**n**	**Model 1**		**Model 2**	
		**IRR**	**95% CI**	**IRR**	**95% CI**
**Comparison: Obesity vs. normal weight[a]**					
A number of NCDs	6,095	1.34	1.22, 1.47***	1.34	1.23, 1.47***
**Comparison: Class II & III obesity vs. normal weight[b]**					
A number of NCDs	6,095	1.53	1.36, 1.72***	1.53	1.36, 1.72***

**p<0.05; **p<0.01; ***p<0.001*

n=analytical sample size (excluding missing observations); IRR=incidence-rate ratio; CI=confidence interval

[a]Comparisons between overweight vs. normal weight are not presented.

[b]Comparisons between overweight vs. normal weight and Class I obesity vs. normal weight are not presented.

Model 1: The association between psychological well-being related measure and a cumulative number of NCDs at follow-up was adjusted for the number of NCDs at baseline, age, sex, ethnicity, marital status, paid employment status, completion of degree qualification level, and wealth index.

Model 2: Model 1 with an additional adjustment for an index of overall psychological well-being.

Index of overall psychological well-being was developed by re-standardizing the average standardized scores of five psychological well-being related measures (depressive symptoms, enjoyment of life, eudemonic well-being, life satisfaction, and loneliness).

Table B16. Longitudinal associations between psychological well-being related measures and the cumulative incidence of NCDs reported from healthcare administrative data in ELSA

	n	Model 1		Model 2	
		IRR	95% CI	IRR	95% CI
Depressive symptoms	6,092	1.03	0.99, 1.06	1.03	0.99, 1.07
Enjoyment of life	6,088	0.98	0.94, 1.01	0.97	0.94, 1.01
Eudemonic well-being	6,083	0.98	0.94, 1.01	0.98	0.94, 1.01
Life satisfaction	6,033	1.00	0.97, 1.04	1.00	0.97, 1.04
Loneliness	6,074	1.01	0.97, 1.04	1.01	0.97, 1.04
Social support	6,087	0.98	0.95, 1.01	0.98	0.95, 1.02
Social strain	6,086	1.02	0.99, 1.06	1.02	0.98, 1.05
Index of overall psychological well-being	6,095	1.02	0.98, 1.06	1.02	0.98, 1.06

**p<0.05; **p<0.01; ***p<0.001*
n=analytical sample size (excluding missing observations); IRR=incidence-rate ratio; CI=confidence interval
Model 1: The association between psychological well-being related measure and a cumulative number of NCDs at follow-up was adjusted for the number of NCDs at baseline, age, sex, ethnicity, marital status, paid employment status, completion of degree qualification level, and wealth index.
Model 2: Model 1 with an additional adjustment for obesity status.
Index of overall psychological well-being was developed by re-standardizing the average standardized scores of five psychological well-being related measures (depressive symptoms, enjoyment of life, eudemonic well-being, life satisfaction, and loneliness).

Table B17. Longitudinal associations between changes in BMI (between Waves 4 and 6) and the incidence of NCDs reported from healthcare administrative data in ELSA

Outcomes	BMI changes		
	**n**	**HR**	**95% CI**
Hypertension	1,512	1.05	0.87, 1.26
Heart disease	3,395	1.00	0.92, 1.08
Stroke	4,452	1.07	0.95, 1.19
Diabetes	3,940	1.03	0.83, 1.28
Arthritis	2,539	1.21	0.94, 1.57
Cancer	3,605	0.98	0.94, 1.02
Memory-related disease	4,634	0.96	0.90, 1.04

**p<0.05; **p<0.01; ***p<0.001*

n=analytical sample size (excluding participants with the outcome before and up to Wave 6 and missing observations); HR=hazard ratio; CI=confidence interval

All the associations were adjusted for age, sex, ethnicity, marital status, paid employment status, completion of degree qualification level, and wealth index.

Table B18. Interaction analyses between obesity and an index of overall psychological well-being in predicting NCDs in ELSA

**Outcomes**	**Reference group: Normal weight * index of overall psychological well-being**				
	**n**	**Model 1**		**Model 2**	
		**HR**	**95% CI**	**HR**	**95% CI**
Hypertension	2,699	0.97	0.80, 1.17	1.03	0.81, 1.30
Heart disease	5,082	1.12	0.94, 1.33	1.10	0.90, 1.34
Stroke	6,181	1.04	0.77, 1.40	1.10	0.71, 1.69
Diabetes	5,629	1.06	0.77, 1.46	0.89	0.61, 1.29
Arthritis	3,929	0.99	0.81, 1.22	1.03	0.78, 1.36
Cancer	5,870	0.81	0.65, 1.01	0.80	0.60, 1.05
Memory-related disease	6,376	1.44	1.12, 1.85**	1.39	0.98, 1.96

**p<0.05; **p<0.01; ***p<0.001*
n=analytical sample size (excluding participants with the outcome before and at baseline and missing observations);
All the interaction analyses were adjusted for age, sex, ethnicity, marital status, paid employment status, completion of degree qualification level, wealth index, obesity, and index of overall psychological well-being related measures.
*Model 1: for obesity * index of overall psychological well-being related measures (comparisons between overweight vs. normal weight are not presented)*
*Model 2: for Class II & III obesity * index of overall psychological well-being related measures (comparisons between overweight vs. normal weight and between Class I obesity vs. normal weight are not presented).*
Index of overall psychological well-being was developed by re-standardizing the average standardized scores of five psychological well-being related measures (depressive symptoms, enjoyment of life, eudemonic well-being, life satisfaction, and loneliness).

Findings from the Health and Retirement Study (HRS)

Table C1. Longitudinal associations between Class II & III obesity (vs. normal weight) and the incidence of NCDs in HRS

**Outcomes**	**Class II & III obesity vs. normal weight**				
	**n**	**Model 1**		**Model 2**	
		**HR**	**95% CI**	**HR**	**95% CI**
Hypertension	2,773	1.39	1.06, 1.83*	1.38	1.05, 1.82*
Heart disease	7,443	1.64	1.35, 2.00***	1.62	1.33, 1.97***
Stroke	9,440	1.34	1.01, 1.79*	1.31	0.98, 1.75
Diabetes	7,594	3.15	2.44, 4.08***	3.14	2.42, 4.06***
Arthritis	3,486	1.59	1.27, 2.00***	1.62	1.29, 2.03***
Cancer	8,609	1.19	0.93, 1.52	1.18	0.92, 1.51
Memory-related disease	9,462	0.66	0.53, 0.83***	0.63	0.51, 0.79***

**p<0.05; **p<0.01; ***p<0.001*

n=analytical sample size (excluding participants with the outcome before and at baseline and missing observations); HR=hazard ratio; CI=confidence interval

Model 1: The association between obesity and an NCD was adjusted for age, sex, ethnicity, marital status, employment status, the number of years in education, and wealth index.

Model 2: Model 1 with an additional adjustment for an index of overall psychological well-being.

Index of overall psychological well-being was developed by re-standardizing the average standardized scores of 10 psychological well-being related measures (depressive symptoms, life satisfaction, loneliness, positive affect, negative affect, purpose in life, anxiety, hopelessness, pessimism, and personal constraint).

Comparisons between overweight vs. normal weight and between Class I obesity vs. normal weight are not presented.

Table C2. Longitudinal associations between psychological well-being related measures and the incidence of NCDs in HRS

	n	Model 1		Model 2	
		HR	95% CI	HR	95% CI
**Outcome: Hypertension**					
Depressive symptoms	2,773	1.07	0.99, 1.16	1.08	0.99, 1.16
Life satisfaction	2,754	0.96	0.89, 1.04	0.96	0.89, 1.04
Loneliness	2,751	0.99	0.91, 1.07	0.99	0.92, 1.08
Social support	2,767	0.97	0.90, 1.05	0.97	0.90, 1.04
Social strain	2,766	1.06	0.98, 1.15	1.05	0.97, 1.14
Positive affect	2,758	0.93	0.86, 1.01	0.94	0.87, 1.01
Negative affect	2,761	1.05	0.98, 1.14	1.05	0.98, 1.14
Purpose in life	2,739	0.97	0.90, 1.06	0.98	0.91, 1.06
Anxiety	2,756	1.05	0.97, 1.14	1.05	0.97, 1.14
Hopelessness	2,758	1.00	0.92, 1.08	0.99	0.91, 1.08
Optimism	2,748	0.98	0.91, 1.06	0.98	0.91, 1.05
Pessimism	2,764	1.01	0.93, 1.09	1.00	0.93, 1.09
Cynical hostility	2,695	1.02	0.94, 1.11	1.01	0.93, 1.09
Personal constraint	2,764	1.07	0.99, 1.16	1.07	0.99, 1.16
Mastery	2,762	0.98	0.91, 1.06	0.98	0.90, 1.06
**Outcome: Heart disease**					
Depressive symptoms	7,443	1.13	1.06, 1.21***	1.13	1.06, 1.21***
Life satisfaction	7,380	0.86	0.80, 0.92***	0.86	0.80, 0.92***
Loneliness	7,359	1.08	1.01, 1.16*	1.08	1.01, 1.16*
Social support	7,423	0.92	0.86, 0.98*	0.92	0.86, 0.98*
Social strain	7,419	1.04	0.97, 1.11	1.03	0.97, 1.11
Positive affect	7,370	0.95	0.89, 1.01	0.95	0.89, 1.01
Negative affect	7,373	1.10	1.03, 1.17**	1.10	1.03, 1.17**
Purpose in life	7,325	0.96	0.90, 1.02	0.96	0.90, 1.02
Anxiety	7,354	1.14	1.07, 1.22***	1.14	1.07, 1.23***
Hopelessness	7,398	1.08	1.01, 1.16*	1.08	1.01, 1.15*
Optimism	7,353	0.95	0.89, 1.01	0.95	0.89, 1.01
Pessimism	7,350	1.09	1.02, 1.17**	1.09	1.02, 1.16**
Cynical hostility	7,229	1.02	0.95, 1.09	1.01	0.95, 1.09
Personal constraint	7,391	1.09	1.03, 1.17**	1.09	1.03, 1.16**
Mastery	7,394	0.95	0.89, 1.01	0.95	0.89, 1.01
**Outcome: Stroke**					
Depressive symptoms	9,440	1.16	1.07, 1.26***	1.16	1.07, 1.26***
Life satisfaction	9,361	0.84	0.77, 0.92***	0.84	0.77, 0.92***
Loneliness	9,339	1.15	1.05, 1.27**	1.15	1.05, 1.27**
Social support	9,416	0.92	0.84, 0.99*	0.92	0.84, 0.99*
Social strain	9,411	1.16	1.06, 1.26**	1.16	1.06, 1.26**
Positive affect	9,356	0.92	0.84, 0.99*	0.92	0.84, 0.99*
Negative affect	9,359	1.20	1.11, 1.30***	1.20	1.11, 1.30***
Purpose in life	9,292	0.85	0.78, 0.92***	0.85	0.78, 0.92***
Anxiety	9,331	1.25	1.14, 1.37***	1.25	1.15, 1.37***
Hopelessness	9,389	1.19	1.09, 1.31***	1.19	1.09, 1.31***
Optimism	9,339	0.93	0.85, 1.01	0.93	0.85, 1.01
Pessimism	9,339	1.18	1.08, 1.28***	1.17	1.07, 1.28***
Cynical hostility	9,175	1.14	1.03, 1.25*	1.13	1.03, 1.25*
Personal constraint	9,371	1.15	1.05, 1.25**	1.15	1.05, 1.25**
Mastery	9,377	0.95	0.87, 1.03	0.95	0.87, 1.03

	n	Model 1		Model 2	
		HR	95% CI	HR	95% CI
**Outcome: Diabetes**					
Depressive symptoms	7,594	1.11	1.03, 1.19**	1.11	1.03, 1.19**
Life satisfaction	7,529	0.94	0.87, 1.01	0.94	0.88, 1.02
Loneliness	7,520	1.00	0.92, 1.08	1.00	0.93, 1.08
Social support	7,579	0.98	0.91, 1.06	0.99	0.92, 1.06
Social strain	7,578	1.11	1.03, 1.19**	1.09	1.01, 1.18*
Positive affect	7,519	0.96	0.89, 1.04	0.96	0.89, 1.04
Negative affect	7,526	1.04	0.97, 1.12	1.04	0.97, 1.12
Purpose in life	7,473	0.98	0.90, 1.07	0.99	0.91, 1.07
Anxiety	7,510	1.07	1.00, 1.16	1.08	1.00, 1.17*
Hopelessness	7,554	1.05	0.97, 1.14	1.04	0.96, 1.13
Optimism	7,516	1.08	1.01, 1.17*	1.08	1.00, 1.17*
Pessimism	7,511	1.07	0.99, 1.15	1.06	0.98, 1.14
Cynical hostility	7,385	1.02	0.94, 1.11	1.01	0.93, 1.09
Personal constraint	7,545	1.05	0.97, 1.13	1.05	0.97, 1.13
Mastery	7,545	0.99	0.92, 1.06	0.99	0.91, 1.06
**Outcome: Arthritis**					
Depressive symptoms	3,486	1.23	1.14, 1.32***	1.23	1.14, 1.32***
Life satisfaction	3,461	0.85	0.79, 0.91***	0.85	0.79, 0.91***
Loneliness	3,456	1.12	1.04, 1.21**	1.12	1.04, 1.21**
Social support	3,477	0.95	0.88, 1.01	0.95	0.88, 1.02
Social strain	3,476	1.16	1.08, 1.24***	1.15	1.07, 1.24***
Positive affect	3,463	0.86	0.80, 0.93***	0.87	0.81, 0.93***
Negative affect	3,465	1.17	1.09, 1.26***	1.18	1.09, 1.26***
Purpose in life	3,439	0.89	0.82, 0.96**	0.88	0.82, 0.95**
Anxiety	3,448	1.16	1.08, 1.27***	1.18	1.09, 1.27***
Hopelessness	3,468	1.17	1.08, 1.26***	1.17	1.09, 1.26***
Optimism	3,452	0.94	0.88, 1.01	0.94	0.87, 1.01
Pessimism	3,452	1.14	1.06, 1.23***	1.14	1.06, 1.23***
Cynical hostility	3,388	1.08	1.00, 1.16*	1.08	0.99, 1.16
Personal constraint	3,465	1.06	0.98, 1.14	1.06	0.98, 1.14
Mastery	3,473	0.96	0.89, 1.02	0.95	0.89, 1.02
**Outcome: Cancer**					
Depressive symptoms	8,609	1.05	0.97, 1.14	1.05	0.97, 1.13
Life satisfaction	8,531	0.94	0.87, 1.01	0.94	0.87, 1.01
Loneliness	8,509	1.02	0.94, 1.10	1.02	0.94, 1.10
Social support	8,585	0.99	0.92, 1.07	0.99	0.92, 1.07
Social strain	8,581	0.98	0.91, 1.06	0.98	0.91, 1.06
Positive affect	8,519	0.95	0.89, 1.03	0.96	0.89, 1.03
Negative affect	8,526	0.99	0.91, 1.07	0.99	0.91, 1.06
Purpose in life	8,473	1.00	0.93, 1.08	1.01	0.93, 1.08
Anxiety	8,497	1.00	0.92, 1.08	1.00	0.92, 1.08
Hopelessness	8,562	1.08	1.00, 1.17*	1.08	0.99, 1.17
Optimism	8,513	0.98	0.92, 1.06	0.98	0.92, 1.06
Pessimism	8,509	1.07	0.99, 1.16	1.07	0.99, 1.16
Cynical hostility	8,359	1.01	0.94, 1.09	1.01	0.93, 1.09
Personal constraint	8,546	0.99	0.92, 1.07	0.99	0.92, 1.07
Mastery	8,550	0.98	0.91, 1.05	0.98	0.91, 1.05

	n	Model 1		Model 2	
		HR	95% CI	HR	95% CI
**Outcome: Memory-related disease**					
Depressive symptoms	9,462	1.28	1.20, 1.36***	1.28	1.21, 1.36***
Life satisfaction	9,386	0.88	0.82, 0.94**	0.89	0.83, 0.95**
Loneliness	9,368	1.27	1.19, 1.36***	1.28	1.20, 1.37***
Social support	9,442	0.92	0.86, 0.99*	0.92	0.86, 0.99*
Social strain	9,438	1.19	1.11, 1.27***	1.19	1.11, 1.28***
Positive affect	9,378	0.83	0.78, 0.89***	0.83	0.77, 0.89***
Negative affect	9,379	1.21	1.13, 1.30***	1.21	1.13, 1.30***
Purpose in life	9,322	0.82	0.77, 0.88***	0.82	0.76, 0.87***
Anxiety	9,359	1.31	1.22, 1.41***	1.31	1.22, 1.41***
Hopelessness	9,412	1.27	1.19, 1.36***	1.28	1.20, 1.37***
Optimism	9,367	0.88	0.83, 0.94***	0.88	0.83, 0.94***
Pessimism	9,363	1.25	1.17, 1.34***	1.26	1.18, 1.35***
Cynical hostility	9,205	1.18	1.09, 1.27***	1.19	1.11, 1.28***
Personal constraint	9,393	1.27	1.20, 1.35***	1.27	1.20, 1.35***
Mastery	9,398	0.90	0.85, 0.96**	0.90	0.85, 0.96**

**p<0.05; **p<0.01; ***p<0.001*
n=analytical sample size (excluding participants with the outcome before and at baseline and missing observations); HR=hazard ratio; CI=confidence interval
Model 1: The association between psychological well-being related measure and an NCD was adjusted for age, sex, ethnicity, marital status, employment status, the number of years in education, and wealth index.
Model 2: Model 1 with an additional adjustment for obesity status.

Table C3. Cross-sectional associations between obesity and psychological well-being related measures in HRS

**Outcomes**	**n**	**Obesity vs. normal weight[a]**		**Class II & III obesity vs. normal weight[b]**	
		**β**	**95% CI**	**β**	**95% CI**
Depressive symptoms	10,860	0.02	-0.03, 0.08	0.08	0.01, 0.15*
Life satisfaction	10,742	-0.02	-0.08, 0.03	-0.07	-0.14, 0.01
Loneliness	10,677	0.05	-0.02, 0.10	0.14	0.06, 0.21***
Social support	10,816	-0.07	-0.13, -0.02*	-0.13	-0.21, -0.06**
Social strain	10,807	0.09	0.04, 0.15**	0.14	0.07, 0.22***
Positive affect	10,684	-0.04	-0.10, 0.01	-0.08	-0.15, -0.01*
Negative affect	10,691	-0.02	-0.08, 0.04	0.01	-0.07, 0.08
Purpose in life	10,629	-0.06	-0.12, -0.01*	-0.11	-0.18, -0.04**
Anxiety	10,682	0.01	-0.05, 0.06	0.04	-0.03, 0.12
Hopelessness	10,746	0.06	0.01, 0.12*	0.13	0.06, 0.20***
Optimism	10,688	0.01	-0.05, 0.07	0.01	-0.07, 0.08
Pessimism	10,676	0.09	0.03, 0.15**	0.19	0.12, 0.26***
Cynical hostility	10,490	0.17	0.11, 0.22***	0.24	0.17, 0.30***
Perceived constraint	10,726	0.01	-0.04, 0.07	0.04	-0.03, 0.11
Perceived mastery	10,732	0.03	-0.03, 0.09	-0.01	-0.08, 0.06
Index of psychological distress	10,802	0.05	-0.01, 0.10	0.13	0.05, 0.20**

**p<0.05; **p<0.01; ***p<0.001*
n=analytical sample size (excluding missing observations); β=regression coefficient; CI=confidence interval
[a]Comparisons between overweight vs. normal weight are not presented.
[b]Comparisons between overweight vs. normal weight and Class I obesity vs. normal weight are not presented.
All the associations were adjusted for age, sex, ethnicity, marital status, employment status, the number of years in education, and wealth index.
Index of overall psychological well-being was developed by re-standardizing the average standardized scores of 10 psychological well-being related measures (depressive symptoms, life satisfaction, loneliness, positive affect, negative affect, purpose in life, anxiety, hopelessness, pessimism, and personal constraint).

Table C4. Mediation by psychological well-being related measures in the longitudinal associations between obesity and the incidence of NCDs in HRS

	**Mediation by psychological well-being related measures**					
	**Obesity vs. normal weight**			**Class II & III obesity vs. normal weight**		
	**n**	**Estimate**	**95% CI**	**n**	**Estimate**	**95% CI**
**Outcome: Heart disease**						
Pessimism	4,678	0.012	-0.017, 0.040	2,794	0.001	-0.036, 0.036
**Outcome: Diabetes**						
Social strain	4,675	0.001	-0.002, 0.003	2,902	-0.002	-0.007, 0.004
**Outcome: Arthritis**						
Loneliness	*NA*			1,295	0.006	-0.036, 0.047
Social strain	2,132	0.014	-0.016, 0.044	1,307	0.008	-0.016, 0.033
Purpose in life	*NA*			1,288	-0.017	-0.066, 0.033
Hopelessness	*NA*			1,302	-0.017	-0.073, 0.038
Pessimism	2,114	0.007	-0.031, 0.046	1,295	0.024	-0.027, 0.076

**p<0.05; **p<0.01; ***p<0.001*
n=analytical sample size (excluding participants with the outcome before and at baseline and missing observations); Estimate=the overall proportion due to mediation or the proportion mediated; CI=confidence interval; ***NA=not applicable*** *(e.g., no significant differences in standardised scores of loneliness, purpose in life, and hopelessness between participants with obesity vs. normal weight at p<0.01 – See Table C3)*
All the mediation analyses were adjusted for age, sex, ethnicity, marital status, employment status, the number of years in education, and wealth index.
As only a binary or continuous independent variable is allowed, causal mediation analysis excluded participants with overweight for the comparison between obesity vs. normal weight, and participants with overweight and Class I obesity for the comparison between Class II & III obesity vs. normal weight, resulting in a smaller analytical sample size compared to previous regression analyses that allowed an ordinal independent variable to be fitted.
See Tables 4 (main document), C1, C2, and C3 to determine whether preconditions for successful mediation were met.

Table C5. Longitudinal associations between obesity and the cumulative incidence of NCDs in HRS

Outcomes	n	Model 1		Model 2	
		IRR	95% CI	IRR	95% CI
**Comparison: Obesity vs. normal weight[a]**					
A number of NCDs	10,217	1.13	1.11, 1.16***	1.14	1.11, 1.16***
**Comparison: Class II & III obesity vs. normal weight[b]**					
A number of NCDs	10,217	1.15	1.12, 1.19***	1.15	1.12, 1.19***

**p<0.05; **p<0.01; ***p<0.001*

n=analytical sample size (excluding missing observations); IRR=incidence-rate ratio; CI=confidence interval

[a]Comparisons between overweight vs. normal weight are not presented.

[b]Comparisons between overweight vs. normal weight and Class I obesity vs. normal weight are not presented.

Model 1: The association between psychological well-being related measure and a cumulative number of NCDs at follow-up was adjusted for the number of NCDs at baseline, age, sex, ethnicity, marital status, employment status, the number of years in education, and wealth index.

Model 2: Model 1 with an additional adjustment for an index of overall psychological well-being.

Index of overall psychological well-being was developed by re-standardizing the average standardized scores of 10 psychological well-being related measures (depressive symptoms, life satisfaction, loneliness, positive affect, negative affect, purpose in life, anxiety, hopelessness, pessimism, and personal constraint).

Table C6. Longitudinal associations between psychological well-being related measures and the cumulative incidence of NCDs in HRS

	n	Model 1		Model 2	
		IRR	95% CI	IRR	95% CI
Depressive symptoms	10,217	1.01	1.00, 1.02*	1.01	1.00, 1.02**
Life satisfaction	10,124	0.99	0.98, 0.99**	0.99	0.98, 0.99**
Loneliness	10,104	1.01	1.00, 1.02	1.01	1.00, 1.02
Social support	10,190	0.99	0.98, 1.00*	0.99	0.98, 1.00*
Social strain	10,185	1.01	1.00, 1.02**	1.01	1.00, 1.02**
Positive affect	10,112	0.99	0.98, 1.00*	0.99	0.98, 1.00*
Negative affect	10,119	1.01	1.00, 1.02*	1.01	1.00, 1.02*
Purpose in life	10,050	1.00	0.99, 1.01	1.00	0.99, 1.01
Anxiety	10,094	1.01	1.01, 1.02**	1.01	1.01, 1.03**
Hopelessness	10,160	1.02	1.01, 1.02**	1.02	1.01, 1.02**
Optimism	10,102	1.00	0.99, 1.01	1.00	0.99, 1.01
Pessimism	10,097	1.02	1.01, 1.02***	1.02	1.01, 1.02**
Cynical hostility	9,919	1.01	1.00, 1.02	1.01	1.00, 1.02
Personal constraint	10,140	1.00	0.99, 1.01	1.00	1.00, 1.01
Mastery	10,141	1.00	0.99, 1.01	1.00	0.99, 1.01
Index of overall psychological well-being	10,217	1.02	1.01, 1.03***	1.02	1.01, 1.03***

**p<0.05; **p<0.01; ***p<0.001*
n=analytical sample size (excluding missing observations); IRR=incidence-rate ratio; CI=confidence interval
Model 1: The association between psychological well-being related measure and a cumulative number of NCDs at follow-up was adjusted for <u>the number of NCDs at baseline,</u> age, sex, ethnicity, marital status, employment status, the number of years in education, and wealth index.
Model 2: Model 1 with an additional adjustment for <u>obesity status</u>.
Index of overall psychological well-being was developed by re-standardizing the average standardized scores of 10 psychological well-being related measures (depressive symptoms, life satisfaction, loneliness, positive affect, negative affect, purpose in life, anxiety, hopelessness, pessimism, and personal constraint).

Table C7. Mediation by psychological well-being related measures in the longitudinal association between obesity and the cumulative incidence of NCDs in HRS

	**Mediation by psychological well-being related measures**					
	**Obesity vs. normal weight**			**Class II & III obesity vs. normal weight**		
	**n**	**Estimate**	**95% CI**	**n**	**Estimate**	**95% CI**
**Outcome: a cumulative number of NCDs**						
Social strain	6,511	-0.001	-0.008, 0.006	3,914	0.001	-0.008, 0.009
Hopelessness	*NA*			3,880	0.011	-0.010, 0.031
Pessimism	6,456	0.002	-0.004, 0.008	3,880	0.011	-0.010, 0.031
Index of overall psychological well-being	*NA*			3,927	-0.000	-0.005, 0.004

**p<0.05; **p<0.01; ***p<0.001*

n=analytical sample size (excluding missing observations); Estimate=the overall proportion due to mediation or the proportion mediated; CI=confidence interval; ***NA=not applicable*** *(e.g., no significant differences in standardised scores of hopelessness and index of overall psychological well-being between participants with obesity vs. normal weight at p<0.01 – See Table C3)*

All the mediation analyses were adjusted for <u>the number of NCDs at baseline</u>, age, sex, ethnicity, marital status, employment status, the number of years in education, and wealth index.

Index of overall psychological well-being was developed by re-standardizing the average standardized scores of 10 psychological well-being related measures (depressive symptoms, life satisfaction, loneliness, positive affect, negative affect, purpose in life, anxiety, hopelessness, pessimism, and personal constraint).

As only a binary or continuous independent variable is allowed, causal mediation analysis excluded participants with overweight and Class I obesity for the comparison between Class II & III obesity vs. normal weight, resulting in a smaller analytical sample size compared to previous regression analyses that allowed an ordinal independent variable to be fitted.

<u>See Tables C3, C5, and C6 to determine whether preconditions for successful mediation were met.</u>

Table C8. Longitudinal associations between psychological well-being related measures and changes in BMI (between Waves 9–10 and 11–12) in HRS

Independent variables	BMI changes		
	n	β	95% CI
Depressive symptoms	8,275	-0.05	-0.15, 0.06
Life satisfaction	8,203	-0.01	-0.11, 0.10
Loneliness	8,177	-0.04	-0.14, 0.06
Social support	8,251	0.03	-0.05, 0.12
Social strain	8,245	-0.13	-0.23 -0.04**
Positive affect	8,178	0.04	-0.05, 0.13
Negative affect	8,186	0.02	-0.08, 0.12
Purpose in life	8,143	0.06	-0.03, 0.16
Anxiety	8,175	0.01	-0.11, 0.12
Hopelessness	8,214	-0.08	-0.18, 0.01
Optimism	8,167	-0.03	-0.12, 0.07
Pessimism	8,169	-0.04	-0.13, 0.05
Cynical hostility	8,035	-0.03	-0.13, 0.06
Personal constraint	8,200	-0.04	-0.13, 0.05
Mastery	8,197	-0.01	-0.11, 0.10
Index of overall psychological well-being	8,250	-0.05	-0.16, 0.06

**p<0.05; **p<0.01; ***p<0.001*

n=analytical sample size (excluding missing observations); β=regression coefficient; CI=confidence interval

All the associations were adjusted for age, sex, ethnicity, marital status, employment status, the number of years in education, and wealth index.

Index of overall psychological well-being was developed by re-standardizing the average standardized scores of 10 psychological well-being related measures (depressive symptoms, life satisfaction, loneliness, positive affect, negative affect, purpose in life, anxiety, hopelessness, pessimism, and personal constraint).

Table C9. Longitudinal associations between changes in BMI (between Waves 9–10 and 11–12) and the incidence of NCDs in HRS

**Outcomes**	**BMI changes**		
	**n**	**HR**	**95% CI**
Hypertension	1,727	0.96	0.92, 1.01
Heart disease	5,243	0.98	0.96, 1.01
Stroke	6,965	1.00	0.96, 1.04
Diabetes	5,247	0.99	0.95, 1.04
Arthritis	2,224	1.03	0.99, 1.07
Cancer	6,234	1.00	0.96, 1.03
Memory-related disease	6,888	0.97	0.95, 1.00*

**p<0.05; **p<0.01; ***p<0.001*

n=analytical sample size (excluding participants with the outcome before and up to Waves 11-12 and missing observations); HR=hazard ratio; CI=confidence interval

All the associations were adjusted for age, sex, ethnicity, marital status, employment status, the number of years in education, and wealth index.

Table C10. Interaction analyses between obesity and an index of overall psychological well-being in predicting NCDs in HRS

**Outcomes**	**Reference group: Normal weight * index of overall psychological well-being**				
	**n**	**Model 1**		**Model 2**	
		**HR**	**95% CI**	**HR**	**95% CI**
Hypertension	2,773	0.97	0.79, 1.19	0.78	0.59, 1.02
Heart disease	7,443	0.96	0.81, 1.13	0.88	0.73, 1.07
Stroke	9,440	1.07	0.86, 1.32	1.05	0.82, 1.33
Diabetes	7,594	1.00	0.81, 1.24	1.03	0.81, 1.32
Arthritis	3,486	0.96	0.80, 1.16	1.07	0.85, 1.35
Cancer	8,609	1.03	0.85, 1.24	1.03	0.81, 1.29
Memory-related disease	9,462	1.14	0.97, 1.35	1.28	1.05, 1.56*

**p<0.05; **p<0.01; ***p<0.001*
n=analytical sample size (excluding participants with the outcome before and at baseline and missing observations); All the interaction analyses were adjusted for age, sex, ethnicity, marital status, employment status, the number of years in education, wealth index, obesity, and index of overall psychological well-being related measures.
*Model 1: for obesity * index of overall psychological well-being related measures (comparisons between overweight vs. normal weight are not presented)*
*Model 2: for Class II & III obesity * index of overall psychological well-being related measures (comparisons between overweight vs. normal weight and between Class I obesity vs. normal weight are not presented).*
Index of overall psychological well-being was developed by re-standardizing the average standardized scores of 10 psychological well-being related measures (depressive symptoms, life satisfaction, loneliness, positive affect, negative affect, purpose in life, anxiety, hopelessness, pessimism, and personal constraint).

Information on planned protocol analyses not reported in the present study

We followed our pre-registered study protocol (https://doi.org/10.17605/OSF.IO/JRQAP) in conducting the analysis. All planned protocol analyses were conducted and reported in the present study, except for additional analyses of metabolic status: i) the associations between obesity and NCD stratified analyses by metabolically health status and ii) metabolically healthy status as an independent variable predicting NCDs. We plan to conduct and then report the analyses focusing on metabolic status in a separate research report given the large number of analyses and different focus of research questions.

References

1. Daly M, Sutin AR, Robinson E. Perceived Weight Discrimination Mediates the Prospective Association Between Obesity and Physiological Dysregulation: Evidence From a Population-Based Cohort. Psychol Sci 2019; 30(7): 1030-1039.
2. Crimmins E, Guyer H, Langa K, Ofstedal M, Wallace R, Weir D. HRS Documentation Report: Documentation of Physical Measures, Anthropometrics and Blood Pressure in the Health and Retirement Study Report. Survey Research Center, University of Michigan: Ann Arbor, MI 2008.
3. Turvey CL, Wallace RB, Herzog R. A revised CES-D measure of depressive symptoms and a DSM-based measure of major depressive episodes in the elderly. International psychogeriatrics 1999; 11(2): 139-48.
4. Steptoe A, Wardle J. Enjoying life and living longer. Arch Intern Med 2012; 172(3): 273-5.
5. Zaninotto P, Wardle J, Steptoe A. Sustained enjoyment of life and mortality at older ages: analysis of the English Longitudinal Study of Ageing. BMJ 2016; 355: i6267.
6. Hyde M, Wiggins RD, Higgs P, Blane DB. A measure of quality of life in early old age: The theory, development and properties of a needs satisfaction model (CASP-19). Aging & Mental Health 2003; 7(3): 186-194.
7. Steptoe A, Demakakos P, de Oliveira C. The psychological well-being, health and functioning of older people in England. The dynamics of ageing 2012: 98.
8. Diener E, Emmons RA, Larsen RJ, Griffin S. The Satisfaction With Life Scale. Journal of Personality Assessment 1985; 49(1): 71-75.
9. Davies K, Maharani A, Chandola T, Todd C, Pendleton N. The longitudinal relationship between loneliness, social isolation, and frailty in older adults in England: a prospective analysis. The Lancet Healthy Longevity 2021; 2(2): e70-e77.
10. Hughes ME, Waite LJ, Hawkley LC, Cacioppo JT. A Short Scale for Measuring Loneliness in Large Surveys: Results From Two Population-Based Studies. Research on aging 2004; 26(6): 655-672.
11. Russell DW. UCLA Loneliness Scale (Version 3): reliability, validity, and factor structure. J Pers Assess 1996; 66(1): 20-40.
12. Khondoker M, Rafnsson SB, Morris S, Orrell M, Steptoe A. Positive and Negative Experiences of Social Support and Risk of Dementia in Later Life: An Investigation Using the English Longitudinal Study of Ageing. Journal of Alzheimer's Disease 2017; 58: 99-108.
13. Watson D, Clark LA. The PANAS-X: Manual for the Positive and Negative Affect Schedule - Expanded Form. University of Iowa, 1994.
14. Ryff CD. Happiness is everything, or is it? Explorations on the meaning of psychological well-being. Journal of personality and social psychology 1989; 57(6): 1069-1081.
15. Ryff CD, Keyes CL. The structure of psychological well-being revisited. J Pers Soc Psychol 1995; 69(4): 719-27.
16. Beck AT, Epstein N, Brown G, Steer RA. An inventory for measuring clinical anxiety: psychometric properties. J Consult Clin Psychol 1988; 56(6): 893-7.
17. Beck AT, Weissman A, Lester D, Trexler L. The measurement of pessimism: The Hopelessness Scale. Journal of Consulting and Clinical Psychology 1974; 42(6): 861-865.
18. Everson SA, Kaplan GA, Goldberg DE, Salonen R, Salonen JT. Hopelessness and 4-year progression of carotid atherosclerosis. The Kuopio Ischemic Heart Disease Risk Factor Study. Arterioscler Thromb Vasc Biol 1997; 17(8): 1490-5.
19. Scheier MF, Carver CS, Bridges MW. Distinguishing optimism from neuroticism (and trait anxiety, self-mastery, and self-esteem): a reevaluation of the Life Orientation Test. J Pers Soc Psychol 1994; 67(6): 1063-78.
20. Cook WW, Medley DM. Proposed hostility and Pharisaic-virtue scales for the MMPI. Journal of Applied Psychology 1954; 38(6): 414-418.
21. Costa PT, Zonderman AB, McCrae RR, Williams RB. Cynicism and paranoid alienation in the Cook and Medley HO Scale. Psychosomatic Medicine 1986; 48(3-4): 283-285.
22. Infurna FJ, Mayer A. The effects of constraints and mastery on mental and physical health: Conceptual and methodological considerations. Psychol Aging 2015; 30(2): 432-448.

23. Putra IGNE, Daly M, Sutin A, Steptoe A, Robinson E. Psychological pathways explaining the prospective association between obesity and physiological dysregulation. Health Psychology 2023.
24. Discacciati A, Bellavia A, Lee JJ, Mazumdar M, Valeri L. Med4way: a Stata command to investigate mediating and interactive mechanisms using the four-way effect decomposition. International Journal of Epidemiology 2019; 48(1): 15-20.
25. Åhlin JK, Halonen JI, Madsen IEH, Rugulies R, Sørensen JK, Magnusson Hanson LL. Interrelationships between job demands, low back pain and depression: A four-way decomposition analysis of direct and indirect effects of job demands through mediation and/or interaction. Journal of Affective Disorders 2021; 282: 219-226.
26. Karlsson IK, Zhan Y, Wang Y, Li X, Jylhävä J, Hägg S, et al. Adiposity and the risk of dementia: mediating effects from inflammation and lipid levels. European Journal of Epidemiology 2022.
27. Burgos Ochoa L, Rijnhart JJM, Penninx BW, Wardenaar KJ, Twisk JWR, Heymans MW. Performance of methods to conduct mediation analysis with time-to-event outcomes. Statistica Neerlandica 2020; 74(1): 72-91.
28. VanderWeele TJ. Mediation Analysis: A Practitioner's Guide. Annual Review of Public Health 2016; 37(1): 17-32.
29. Mansournia MA, Altman DG. Inverse probability weighting. BMJ 2016; 352: i189.
30. Chesnaye NC, Stel VS, Tripepi G, Dekker FW, Fu EL, Zoccali C, et al. An introduction to inverse probability of treatment weighting in observational research. Clinical Kidney Journal 2022; 15(1): 14-20.
31. Bu F, Zaninotto P, Fancourt D. Longitudinal associations between loneliness, social isolation and cardiovascular events. Heart 2020; 106(18): 1394-1399.
32. Konttinen H, van Strien T, Männistö S, Jousilahti P, Haukkala A. Depression, emotional eating and long-term weight changes: a population-based prospective study. International Journal of Behavioral Nutrition and Physical Activity 2019; 16(1): 28.
33. Katsoulis M, Stavola BD, Diaz-Ordaz K, Gomes M, Lai A, Lagiou P, et al. Weight Change and the Onset of Cardiovascular Diseases: Emulating Trials Using Electronic Health Records. Epidemiology 2021; 32(5).
34. Aronow WS. Association of obesity with hypertension. Ann Transl Med 2017; 5(17): 350.
35. Kim MS, Kim WJ, Khera AV, Kim JY, Yon DK, Lee SW, et al. Association between adiposity and cardiovascular outcomes: an umbrella review and meta-analysis of observational and Mendelian randomization studies. European Heart Journal 2021; 42(34): 3388-3403.
36. Shay M, MacKinnon AL, Metcalfe A, Giesbrecht G, Campbell T, Nerenberg K, et al. Depressed mood and anxiety as risk factors for hypertensive disorders of pregnancy: a systematic review and meta-analysis. Psychological Medicine 2020; 50(13): 2128-2140.
37. Pan Y, Cai W, Cheng Q, Dong W, An T, Yan J. Association between anxiety and hypertension: a systematic review and meta-analysis of epidemiological studies. Neuropsychiatr Dis Treat 2015; 11: 1121-30.
38. Riaz H, Khan MS, Siddiqi TJ, Usman MS, Shah N, Goyal A, et al. Association Between Obesity and Cardiovascular Outcomes: A Systematic Review and Meta-analysis of Mendelian Randomization Studies. JAMA Network Open 2018; 1(7): e183788-e183788.
39. Van der Kooy K, van Hout H, Marwijk H, Marten H, Stehouwer C, Beekman A. Depression and the risk for cardiovascular diseases: systematic review and meta analysis. Int J Geriatr Psychiatry 2007; 22(7): 613-26.
40. Gan Y, Gong Y, Tong X, Sun H, Cong Y, Dong X, et al. Depression and the risk of coronary heart disease: a meta-analysis of prospective cohort studies. BMC Psychiatry 2014; 14(1): 371.
41. Pan A, Sun Q, Okereke OI, Rexrode KM, Hu FB. Depression and Risk of Stroke Morbidity and Mortality: A Meta-analysis and Systematic Review. JAMA 2011; 306(11): 1241-1249.
42. Pérez-Piñar M, Ayerbe L, González E, Mathur R, Foguet-Boreu Q, Ayis S. Anxiety disorders and risk of stroke: A systematic review and meta-analysis. European Psychiatry 2017; 41: 102-108.
43. Roy T, Lloyd CE. Epidemiology of depression and diabetes: a systematic review. J Affect Disord 2012; 142 Suppl: S8-21.

44. Kan C, Silva N, Golden SH, Rajala U, Timonen M, Stahl D, et al. A Systematic Review and Meta-analysis of the Association Between Depression and Insulin Resistance. Diabetes Care 2013; 36(2): 480-489.
45. Zheng H, Chen C. Body mass index and risk of knee osteoarthritis: systematic review and meta-analysis of prospective studies. BMJ Open 2015; 5(12): e007568.
46. Crowson CS, Matteson EL, Davis JM, 3rd, Gabriel SE. Contribution of obesity to the rise in incidence of rheumatoid arthritis. Arthritis Care Res (Hoboken) 2013; 65(1): 71-7.
47. Fakra E, Marotte H. Rheumatoid arthritis and depression. Joint Bone Spine 2021; 88(5): 105200.
48. Dobbins M, Decorby K, Choi BC. The Association between Obesity and Cancer Risk: A Meta-Analysis of Observational Studies from 1985 to 2011. ISRN Prev Med 2013; 2013: 680536.
49. Wang Y-H, Li J-Q, Shi J-F, Que J-Y, Liu J-J, Lappin JM, et al. Depression and anxiety in relation to cancer incidence and mortality: a systematic review and meta-analysis of cohort studies. Molecular Psychiatry 2020; 25(7): 1487-1499.
50. Park S-J, Ko H-J, Youn C-H, Kim AS, Moon H, Eun H-J. Obesity and the Risk of Dementia: A Meta-Analysis of Prospective Cohort Studies. KJFP 2018; 8(1): 118-124.
51. Beydoun MA, Beydoun HA, Wang Y. Obesity and central obesity as risk factors for incident dementia and its subtypes: a systematic review and meta-analysis. Obes Rev 2008; 9(3): 204-18.
52. Cherbuin N, Kim S, Anstey KJ. Dementia risk estimates associated with measures of depression: a systematic review and meta-analysis. BMJ Open 2015; 5(12): e008853.
53. Stafford J, Chung WT, Sommerlad A, Kirkbride JB, Howard R. Psychiatric disorders and risk of subsequent dementia: Systematic review and meta-analysis of longitudinal studies. International Journal of Geriatric Psychiatry 2022; 37(5).
54. Atasoy S, Johar H, Peters A, Ladwig K-H. Association of hypertension cut-off values with 10-year cardiovascular mortality and clinical consequences: a real-world perspective from the prospective MONICA/KORA study. European Heart Journal 2019; 40(9): 732-738.
55. Bell JA, Kivimaki M, Hamer M. Metabolically healthy obesity and risk of incident type 2 diabetes: a meta-analysis of prospective cohort studies. Obes Rev 2014; 15(6): 504-15.
56. National Institute for Health and Care Excellence. Type 2 diabetes: prevention in people at high risk. In, 2017.
57. Weden MM, Shih RA, Kabeto MU, Langa KM. Secular Trends in Dementia and Cognitive Impairment of U.S. Rural and Urban Older Adults. American Journal of Preventive Medicine 2018; 54(2): 164-172.
58. Hale JM, Schneider DC, Mehta NK, Myrskylä M. Cognitive impairment in the U.S.: Lifetime risk, age at onset, and years impaired. SSM - Population Health 2020; 11: 100577.
59. Williams BD, Pendleton N, Chandola T. Cognitively stimulating activities and risk of probable dementia or cognitive impairment in the English Longitudinal Study of Ageing. SSM - Population Health 2020; 12: 100656.
60. Davies HR, Cadar D, Herbert A, Orrell M, Steptoe A. Hearing Impairment and Incident Dementia: Findings from the English Longitudinal Study of Ageing. J Am Geriatr Soc 2017; 65(9): 2074-2081.
